# Searching for New Clues about the Molecular Cause of Endomyocardial Fibrosis by Way of *In Silico* Proteomics and Analytical Chemistry

**DOI:** 10.1371/journal.pone.0007420

**Published:** 2009-10-12

**Authors:** Misaki Wayengera

**Affiliations:** Division of Molecular Pathology, Department of Pathology, School of Biomedical Sciences, College of Health Sciences, Makerere University, Kampala, Uganda; Institute of Infectious Disease and Molecular Medicine, South Africa

## Abstract

**Background:**

Endomyocardial Fibrosis (EMF) –is a chronic inflammatory disease of the heart with related pathology to that of late stage Chaga's disease. Indeed, both diseases are thought to result from auto-immune responses against myocardial tissue. As is the case that molecular mimicry between the acidic termini of Trypanosoma *cruzi* ribosomal P0, P1 and P2β (or simply TcP0, TcP1, and TcP2β) proteins and myocardial tissue causes Chaga's disease, excessive exposure to certain infections, toxins including cassava ones, allergy and malnutrition has been suggested as the possible cause for EMF. Recent studies have defined the proteomic characteristics of the T. *cruzi* ribosomal P protein-C-termini involved in mediating auto-immunity against Beta1-adrenergic receptors of the heart in Chaga's disease. This study aimed to investigate the similarity of C-termini of TcP0/TcP2β to sequences and molecules of several plants, microbial, viral and chemical elements- most prior thought to be possible causative agents for EMF.

**Methods and Principal Findings:**

Comparative Sequence alignments and phylogeny using the BLAST-P tool at the Swiss Institute of Biotechnology (SIB) revealed homologs of C-termini of TcP0 and TcP2β among related proteins from several eukaryotes including the animals (Homo *sapiens*, C. *elegans*, D. *melanogaster*), plants (*Arabidopsis thaliana*, *Zea mays*, *Glycina Max*, Oryza sativa, *Rhizopus oryzae*) and protozoa (P. *falciparum*, T. *gondii*, Leishmania *spp*).The chemical formulae of the two T.*cruzi* ribosomal protein C-terminal peptides were found to be C_61_H_83_N_13_O_26_S_1_and C_64_H_87_N_13_O_28_S_1_ respectively by Protparam. Both peptides are heavily negatively charged. Constitutively, both auto-antigens predominantly contain Asparagine (D), Glycine (G) and Phenylamine (F), with a balanced Leucine (L) and Methionine (M) percent composition of 7.7%. The afore going composition, found to be non-homologous to all molecules of chemical species in the databases searched, suggests the possible role of a metabolic pathway in the pathogenesis of EMF if aligned with our “molecular mimicry” hypothesis.

**Conclusions:**

Our findings provide a “window” to suggest that cross reactivity of antibodies against C-terminal sequences of several animal, plant and protozoal ribosomal P proteins with heart tissue may mediate EMF in a similar manner as C- termini of T. *cruzi* do for Chaga's disease.

## Introduction

**Endomyocardial fibrosis or simply EMF is a restrictive cardiomyopathy known to affect persons of defined geographical locales and socioeconomic status** [**1**], [2****] **. First described at the Department of Pathology-Makerere University, Uganda by the Pathologist J.N.P Davies in 1948** [**3**], the important features of this disease—namely, geographical distribution, cardiac specificity and preference for the socioeconomically poor, have evaded a complete scientific explanation despite the intense scientific scrutiny to which the disease has been subjected[4], [5]. Although the pathological lesions in EMF have been clearly found to comprise fibrosis and calcification, possibly resulting from long standing inflammatory responses, no natural insult is evidenced to cause such pathology [5], [6]. Specifically, in as much as several potential insults have been proposed as the primary cause for EMF, including Infection (Toxoplasmosis, Rheumatic fever, Malaria, Myocarditis and Helminthes [7]), allergy (Autoimmunity and Eosinophilia [8]), malnutrition (Protein or Magnesium deficiency[5], [7]) and toxic agents(Cassava, other plant toxins, Arsenic[9], Cerium, Thorium, Serotonin, or Vitamin D[5]); no single one is proven[5], [10]. Existing evidence for an ethnic predisposition points to a possible genetic idiosyncrasy [11], [12]. Largely because of the above lack of evidence for a particular causative insult, the disease remains unpreventable [5]. Recent studies indicate that there might indeed be a decline in the incidence of EMF paralleled to improvement in the socioeconomic welfare of high risk populations [4]. Until now, the only evidenced benefit for drug use in EMF-deterring progression of the inflammatory pathology, has revolved around steroids [13], with the list of trial drugs expanding to include, more lately, serotonin receptor inhibitors [14], [15]. Surgery, mainly that involving cardiomyoectomy of pathological lesions (plus reconstruction of the heart architecture), has a role despite its infrequent use due to poor state of heart surgery available in regions where EMF is similarly prevalent [15]. Ideally, all EMF patients with stage III and IV heart failure would benefit from a heart transplant [15]. The foregoing picture underlines the need to devise novel, cheap and yet still effective medical interventions against EMF.

**Figure 1:**
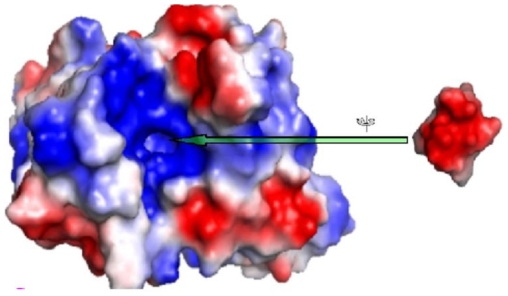
Showing the binding site of the acidic C-termini of the T. *cruzi* ribosomal P proteins on the specific human antibody. The Figure illustrates the unique binding feature conferred by the positive charge within the binding site of the acidic C-termini of the T. *cruzi* ribosomal P proteins (themselves negatively charged) on the specific human antibody. The van der Waals surface is coloured according to the electrostatic potential calculated with the program Poisson-Boltzman electrostatics calculated employing using APBS as implemented in PyMol with default charge settings and dielectric constant 80 (Receptor coloured by calculated charge from red −1 to blue +1). Note that the binding site of the peptides is a positive charged cavity. This work is reminiscent of the recent findings towards a better understanding of the molecular pathology of Chaga's disease, citation [17] Martin OA, Villegas ME, Aguilar CF (2009) Three-dimensional studies of pathogenic peptides from the c-terminal of Trypanosoma cruzi ribosomal P proteins and their interaction with a monoclonal antibody structural model. *PMC Biophys*. 2(1):4.

In the past, the pathophysiology of EMF has been closely related to that of several other cardiomyopathies, including the hypereosinophilic syndrome (Loffler's disease)[15], and Chaga's disease[16]. Specifically, all diseases are known to possess a spectrum of pathology that encompasses hypereosinophilia, fibrosis and or, in long standing cases, calcification [15], [16]. Recent studies have established molecular mimicry as the mechanisms for pathology in some of the above EMF related (particularly Chaga's) cardiomyopathies [16]. Specifically, auto antibodies to the acidic C- termini of two Trypanosoma *cruzi* (or simply T.*cruzi*) ribosomal proteins (TcP0 & TcP2β, respectively: EDDDDDFGMGALF **and** EEEDDDMGFGLFD) have been associated with the chronic cardiac pathology of Chaga's disease in humans [16]. Martin et *al.* [17] have recently described, using 3-dimensional modeling and docking experiments, a more clear interaction of the structural elements involved in the autoimmune mechanism of anti-P auto-antibodies cross-reaction and stimulation of the β1-adrenoreceptor, results that may lead eventually to the development of treatments to abolish receptor mediated symptoms in Chaga's disease**( see** **Figure 1** **for illustration** [**17**] **)**. Given the prior observed related pathology in both diseases, we hypothesized, that molecular mimicry may explain the pathology seen in both diseases too. By so doing, we also sub-hypothesized that the molecular insult in Chaga's disease may bear similarity (used interchangeably with resemblance here to imply analogy and not necessary homology) to the insult responsible for EMF. This study was conducted to examine the specific-hypothesis that resemblance (analogy) between the C-termini of the two T. *cruzi* ribosomal proteins TcP0/TcP2β and prior suspected causative insults for EMF explains the commonality of gross pathology. Initially designed to comprise an initial exploratory *In Silico* phase exploiting comparative sequence alignments [18], [19] and subsequent *In Situ* hybridization proof of concept phase, the herein presented non-specific results of the exploratory phase made it difficult to conduct parallel confirmatory *In Situ* inquiry due to a wide spectrum of test candidates and limited resources. Specifically, contrarily to prior data pointing to an architectural conservation of ribosomal P protein- structure across some life domains, no sequence similarity was found between the acidic termini of T.*cruzi* ribosomal P proteins TcP0/TcP2β and sequences of all searched plant, microbial and viral databases by initial NCBI microbial BLAST-P at default. Repeat BLAST at SIB, however, revealed that both C-termini of T. *cruzi* ribosomal P protein TcP0 and TcP2β exhibit homology to acidic termini of respective eukaryotic proteins. Further, the C-termini of TcP0 and TcP2β are noted to possess characteristic amino acid composition that confer unto them acidity and negative charge. Overall, we provide evidence to suggest that cross reactivity of antibodies against C-terminal sequences of several animal, plant and protozoal ribosomal P proteins with heart tissue may mediate EMF in a similar manner as C- termini of T. *cruzi* do for Chaga's disease. It is, never the less, still possible that the mechanisms of molecular mimicry between the suspected EMF-insults and myocardial tissue are mediated via different myocardial antigens altogether- thereby, making the specified protein-portions in our study not the likely cause of EMF.

## Results

### 1.0 Similarity of C-termini of TcP0 and TcP2β to analyzed pathogen, plant, viral proteins and the human proteome

#### 1.10NCBI BLAST-P at Default and other settings

But for the hits on the source organism's ribosomal proteins (corresponding to the queries: acidic termini of TcP0 and TcP2β [20]–[24]), no similarity was found to proteins of all searched organismal, plant and viral protein genome wide databases (PGDB; **for details see** **Table 1** **and Supporting file S1**), findings that were considered ambiguous in light of prior studies [20]–[24]; and SIB BLAST-P tool[18], [19], [25] generated results discussed below. Specifically, noticeable was that, despite the presence of completed *Toxoplasma* genomes among the microbes and several other protozoa including the pathogens of malaria and leishmaniasis (that have previously been suspected to be potential causative insults for EMF [5]); and particularly ones whose whole length ribosomal proteins P0, P1, and P2 have been phylogenetically related to those of T.*cruzi* [*26]*, [28**], no sequence homology or analogy was found with C-termini of P0, P1, and P2 proteins of over 2, 000 searched pathogens, plants, and viruses species. We found this to be an error of default (discussed further below) prompting us to repeat the alignments using the BLAST-P tool at the Swiss Institute of Biotechnology (SIB).

**Table 1:** Showing the hits in the T.*cruzi Proteome* as the native source of the TcP0 C-terminus queried.

Seq. ID	Sequence Producing significant alignment	Sore (Bits)	E value
gb|EAN99267.1|	60S acidic ribosomal protein P0 [Trypanosoma c…	29.3	2.2
gb|EAN99266.1|	60S acidic ribosomal protein P0 [Trypanosoma c	29.3	2.2

Note that the low score values above do not indicate a lower similarity, but rather the fact that the query only made up a small portion of the entire 60S acidic ribosomal protein P0 of T,*cruzi*. Overall, both hits are 100% homologous to the respective match segments of 60S acidic terminus of the T.*cruzi* ribosomal protein P0.

#### 1.20 SIB BLAST-P at default setting

**Figure 2:**
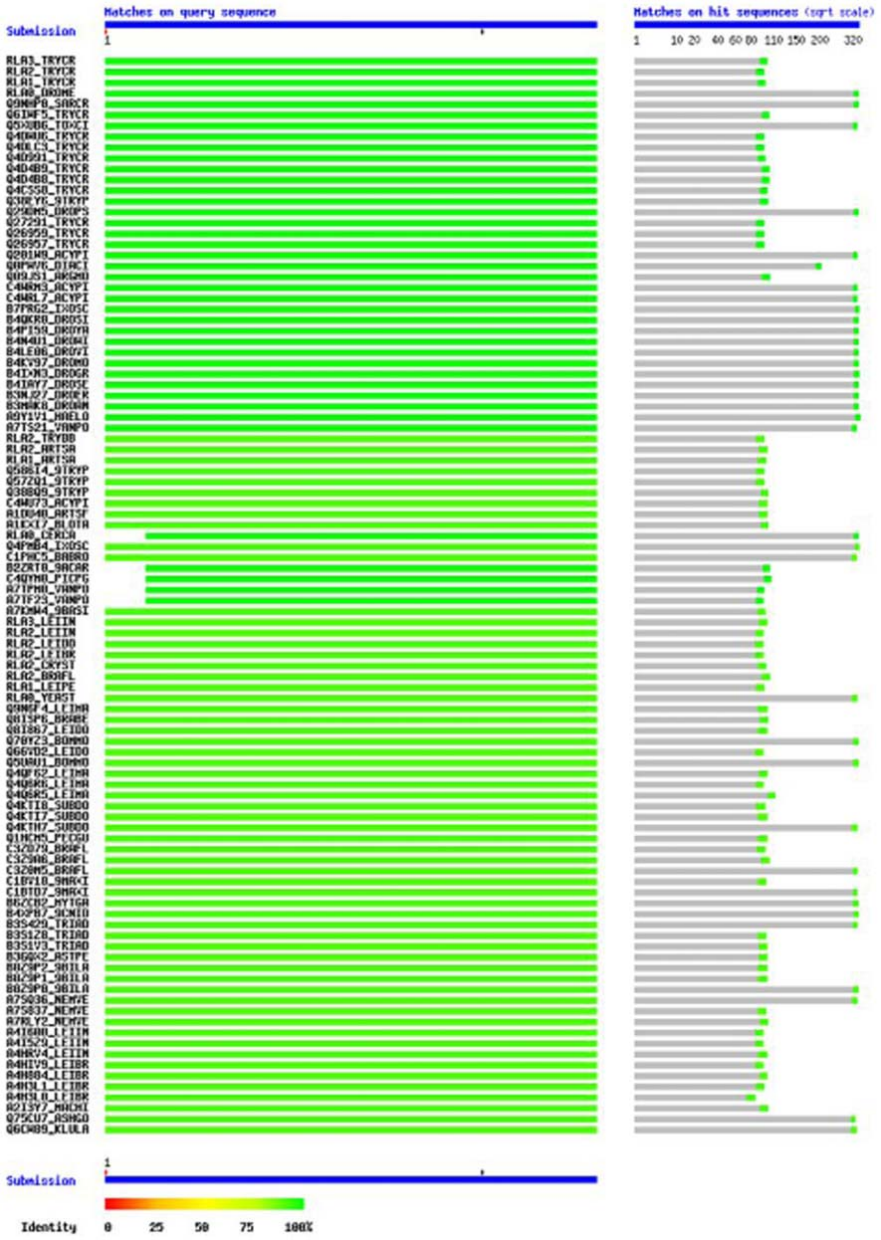
Showing the schematics of the BLAST hits obtained by querying the C-termini of TcP2β across a Swiss Prot database using the BLAST tool at SIB. The Figure illustrates the schematics of the Scores and E values of hits obtained by querying the 13 amino acid sequences of the C-termini of TcP2β (EEEDDDMGFGLFD) across a Swiss Prot database using the BLAST tool at SIB. Note the presence of a key at the bottom to annotate meaning to the colors. Interpretation of this schematics may be best done with table 2 and supplementing file 2 in hand. Briefly, % identity of hits declines as the green color (signifying 100% identity) fades from green to finally red (signifying 0% identity).The data was generated by the BLAST tool at the following URL: SIB availablehttp://www.expasy.ch/cgi-bin/blast.pl

Repeat alignments of acidic termini of TcP0 and TcP2β sequences with sequences of proteins available in the Swiss Prot database using BLAST-P at SIB, contrarily to findings of the NCBI BLAST-P tool above, revealed homologs of both TcP0 and TcP2β acidic termini of eukaryotic origin; including from animals ( Homo *sapiens*, C. *elegans*, D. *melanogaster*), plants(*Arabidopsis thaliana*, *Zea mays*, *Glycina Max*, Oryza sativa, *Rhizopus oryzae*) and protozoal (P. *falciparum*, T. *gondii*, Leishmania *spp*.)**(See** **Table 2** **and Supporting file S2 for detailed results)**. The other species that possess homologous C-termini of P ribosomal proteins to those of T *cruzi* are listed here along with their “common names”: *Toxoptera citricida* aka citrus aphid, *Acyrthosiphon pisum* aka pea aphid, *Argas monolakensis* aka mano lake bird tick, *Diaphorina citri* aka Asian Citrus Phyllid, *Ixodes scapularis* aka Black-legged tick; *Haemaphysalis longicornis* aka Bush tick, *Artemia salina* and *Artemia franciscana* aka Brine shrimp, *Blomia tropicalis* aka Mite, *Ceratitis capitata* aka Mediterranean fruit fly; *Pichia pastorilis* aka yeast, *Saccharomyces cerevisiae* aka Baker's yeast; *Leishmania spp*; *Bombyx mori* aka Silk moth; *Suberites domuncula* aka Sponge, *Asterina pectinifera* a aka Starfish; and *Candida sphaerica*. **Figure 2** shows the schematics of these BLAST hits obtained by querying TcP2B on the Swiss Prot database, **while Figure S1** shows a resultant taxonomic tree from the same search.

**Table 2:** List of Eukaryota spp. with homologous sequences to TcP2B.

Species	spp. Sub-type/Protein where known	% Identity
***Trypanosoma spp.***		**100**
	T.cruzi/P2B	
	T.cruzi/P2A;P-JL5/L12E	
	T.cruzi/P1	
	T.cruzi/P2	
	T.brucei/? P2	
***Drosophila spp.***		**100**
	D.Melanogaster/P0 or DNA-APE	
	D. pseudoobscura pseudoobscura/?	
	D.simulans	
	D. yakuba	
	D. willistoni	
	D. virilis	
	D. mojavensis	
	D. grimshawi	
	D. sechellia	
	D. erecta	
	D. ananassae/P0	
Leishmania spp		92
	L. infantum/P21-LIP	
	L. Donovani/P2	
	L. braziliensis/P2B	
	L. peruviana/P1	
	L. major/P2	
***Others spp***		**>90; ≤100**
	Sarcophaga crassipalpis/P0	100
	Toxoptera citricida/P0	100
	Acyrthosiphon pisum/P0	100
	Diaphorina citri/P0	100
	Ixodes scapularis/P0	100
	Haemaphysalis longicornis/P0	100
	Kluyveromyces polysporus/?	100
	Vanderwaltozyma polysporus/?	100
	Artemia salina/P2	100
	Blomia tropicalis/Blot alt-a6 allergen	100
	Ceratitis capitata/P0	100
	Babesia rodhaini/P0	92
	Pichia pastoris/P2A	92
	Melampsora medusae f. sp. Deltoidis/P1	92
	Branchiostoma floridae-Amphioxus/P2	92
	Saccharomyces cerevisiae/P0	92
	Branchiostoma belcheri-Amphioxus/P1	92
	Bombyx mori/P0	92
	Suberites domuncula-Sponge/P2	92
	Suberites domuncula-Spongeb/L10e/P0	92
	Pectinaria gouldii -Trumpet worm/P1	92
	Lepeophtheirus salmonis-louse/P2, P0	92

Note that some of the hits in Trypanasoma spp., Drosophila spp., and Leishmania spp., -although possessing the indicated identities, have score and E-values that places them at a lower place(for details, see supporting File 2).

### 2.0 Primary structure and chemical composition of the C-terminus of TcP0 and TcP2β

The chemical formulae of the two T.*cruzi* ribosomal P proteins' acid termini were found to be C_61_H_83_N_13_O_26_S_1_and C_64_H_87_N_13_O_28_S_1_ respectively. Details of the amino acid composition, molecular weight, Theoretical PI, Atomic composition, extinction coefficient, Aliphatic Index and hydrophobicity are **shown in Supporting files S3 and S4**. Note that chemically, both peptides may be classified as polycarbonated. Surprisingly, although both C-terminal peptides were noted to contain closely similar amino acid compositions, their respective predicted Instability Indices (II) are differing, with the terminus of TcP0 being stable (at an II value of 11.7) and that of TcP2β unstable (II value of 83.69). Generally, any peptide with an II above 40 is denoted as unstable [25]. These findings-discussed further below, serve to emphasize, how, proteins with closely related amino acid composition may possess differing In V*ivo* biophysical profiles and thus possibly functions as well.

### 2.0 Similarity of the C-terminus of TcP0 and TcP2β to known chemical species

Constitutively, both auto-antigens( acidic termini of TcP0 and TcP2β ) were found to predominantly contain Asparagine(D), Glycine(G) and Phenylamine(F), with a balanced leucine(L) and methionine(M) percent composition of 7.7%**(see Supporting files S3 and S4 for details)**; composition that was found to be non-homologous to any known chemical species to date. Note that this composition is associated with extensive negativity of the peptides- overall charge, uniquely imposed by the amino acids Asparagine and glutamine that are most present within the constitution of both peptides. This is not surprising, since both these terminal peptides are found localized within the acidic C-termini of the T. *cruzi* ribosomal proteins [16], [17]. Specifically, these findings tally with the findings of Martin et *al.* [17]'s 3-dimentional structure of TcP0/TcP2β acidic termini specific human antibody model that exhibits a most remarkable feature in the active site, the positively charged, narrow and deep cavity where these acidic residues 3 to 6 are accommodated, further emphasizing the fact that the most important elements in the molecular peptide recognition by the antibody may be the shape of the loop and the presence of negative charges in positions 3–5 of the acidic peptides P0, P2β [17]. Although rather improbable(and apparently non- evidenced); the notable absence of the positively charged amino acid residues Argenine and Lysine in both the C-terminal sequences of TcP0 and TcP2β prompted us to speculate, whether or not, a deficient diet on Arginine and Lysine could lead to EMF. Several pathways are possible here fore, including: either eliciting a plethora of “systemic effects”(because of their role as precursor or intermediates on several metabolic networks) or having a direct pathological impact on protein biosynthesis (as direct precursor of proteins) [5], [7].

## Discussion

Our findings offer the first ever evidence to support the postulate that cross reactivity of antibodies against C-terminal sequences of ribosomal P proteins from several animals, plant and protozoal with heart tissue may mediate EMF in a similar manner as C- termini of T. *cruzi* do for Chaga's disease. Overall, despite previous studies implicating several factors in the etiology of EMF[5], including the evidenced role of ethnicity [6] and suspicions around Infections (Toxoplasmosis, Rheumatic fever, Malaria, Myocarditis and Helminthes [7]), allergy (Autoimmunity and Eosinophilia [8]), malnutrition (Protein or Magnesium deficiency[5], [7]) and toxic agents(Cassava, other plant toxins, Arsenic[9], Cerium, Thorium, Serotonin, or Vitamin D[5]) as the primary EMF insult; none is yet proven[5], [10]. Collectively, the pathology seen in EMF has been suspected to be mediated via similar molecular mimicry mechanisms as is seen in Loffler's and Chaga's disease [16]. In light of the recent advances towards understanding the mechanism of molecular mimicry seen in Chaga's disease resulting from resemblance of the C-terminal peptides of T.*cruzi* ribosomal P (TcP0 and TcP2β) proteins to cardiac tissue **(see** **Figure 1** **)** [16], [17], we felt it responsive to investigate the potential resemblance (analogy) of the above various suspected insults in EMF to the same (C-termini of T. *cruzi* ribosomal proteins TcP0 and TcP2β). Both P0 and P2 are a major component of the GTPase center of the large ribosomal subunit. The GTPase center, which is located at the N-termini, and functions as a landing platform for translation factors- is regarded as one of the oldest structures in the ribosome and is, presumably, one universally conserved structure in all domains of life [26]–[28]. It has been hypothesized that this structure could indeed be responsible for the major breakthrough on the way to the RNA/protein world, since its appearance would have dramatically increased the rate and accuracy of protein synthesis. Notably, one of the most characteristic ribosomal structures is the stalk: a highly flexible and universal lateral protuberance on the large subunit which is directly involved in the interaction of elongation factors, participating in the translocation mechanism [28]. In eukaryotes the stalk is formed by the pentameric complex P0–(P1)_2_ (P2)_2_ that is reminiscent of the bacterial complex L10–(L7/L12)_4_. In particular, the P0 protein is the eukaryotic L10 equivalent and has a key role in the stalk structure[z]. Interestingly, prior studies have actually pointed to conservation of ribosomal proteins from species within the related life Domain [26], [27]. Functionally, these proteins bind to the highly conserved 26S/28S rRNA GTPase center through the N-terminal domain [28] at sites that are equivalent to those found in bacteria [26], [28]. The P0 C-terminal domain, in particular, is known to interact with the acidic phosphoproteins P1 and P2 (the L7/L12 equivalents) through their N-terminal domains, forming the tip of the stalk [**28**]. The main functional part of the stalk in all domains of life is composed of small L12/P proteins- and these have, until now, been believed to form an evolutionarily conserved group in all species. We show in **Tables 1** **and** **2** *plus* **Supporting files S1 and S2**, that although no sequence similarity was found between the acidic termini of T.*cruzi* ribosomal P proteins TcP0/TcP2β and sequences of all searched plant, microbial and viral databases by initial NCBI microbial BLAST-P at default settings, in line with prior data pointing to an architectural conservation of ribosomal P protein- structure across some life domains[26]–[28], repeat alignments using the BLAST-P Software and algorithms at the Swiss Institute of Biotechnology (SIB), revealed homologs of both studied C-termini of TcP0 and TcP2β with ribosomal P proteins (and in one incidence-D. *melanogaste*r: DNA Apurinic apyramidinic endonucleases-APE) of several eukaryotes including the animals ( Homo *sapiens*, C. *elegans*, D. *melanogaster*), plants(*Arabidopsis thaliana*, *Zea mays*, *Glycina Max*, Oryza sativa, *Rhizopus oryzae*) and protozoa (P. *falciparum*, T. *gondii*, Leishmania *spp*. The schematics of those BLAST hits obtained by querying TcP2B on the Swiss Prot database are shown in **Figure 2**, while **Figure S1** shows the taxonomic tree from the same). Grela and colleagues [**28**] recently performed a comprehensive comparative analysis of the L12/P proteins from the three domains of life and found that bacterial and archaeo/eukaryal L12/P-proteins are not structurally related and, therefore, might not be linked evolutionarily either. Consequently, it has been suggested that proteins be regarded as analogous rather than homologous systems and probably appeared on the ribosomal particle in two independent events in the course of evolution [**28**]. Therefore, in as much as prior insights into the structure of the ribosomes and their components at high resolution leaves no question that the overall architecture of the translational machinery of the cell has been strongly conserved in all kingdoms, it is worth noting that inter-kingdom differences among ribosomal components may inevitably exist, even though the functional significance of these structural variations has not been clarified yet [26]–[28]. Overall, our findings of several eukaryotic homologs of T.*cruzi* ribosomal P protein acidic termini provide a “window” to suggest that cross reaction of antibodies against C-terminal sequences of several animal, plant and protozoal ribosomal P proteins with heart tissue possibly mediates EMF in a similar manner as C- termini of T. *cruzi* do for Chaga's disease. It is, nevertheless, equally still likely that the mechanisms of molecular mimicry between prior suspected EMF-insults and myocardial tissue are mediated via different myocardial antigens- thereby, making the specified protein-portions in our study not the likely cause of EMF.

Considering that our “molecular mimicry” hypothesis is affirmed in animal models for EMF as has been for Chaga's[17], one of the major challenges to explore in future will be determining the likely mechanism of exposure to the these, now observed as, eukaryota conserved ribosomal P protein C-termini. We postulate that metabolic uptake may be such one candidate route of exposure to consider. The possibility that metabolic uptake of the still undefined insult plays a role in the aetiology of EMF arises in light of earlier work that had speculated that metabolites of plantain ingestion (specifically 5-hydroxyindolylacetic) may be the cause of EMF [29], [30]. In a latter controlled study of thirty Nigerians with established endomyocardial fibrosis, however, Ojo [31] found that no significant increase in serum 5-hydroxytryptamine levels occurred in these patients after plantain ingestion. This finding underlined the difference between endomyocardial fibrosis and carcinoid heart disease by proving that no correlation exists between the incidence of endomyocardial fibrosis and the high content of 5-hydroxytryptamine in the local dietary staples [31]. Note, however, that it did not rule out the possibility that another nutritional metabolite other than 5-hydroxytryptamine may be the primary insult, a fact that is further underscored here by the characteristic amino acid composition and negative charge orientation of both studied T. *cruzi* ribosomal proteins- TcP0 and TcP2β C-termini in **Supporting files S3 and S4**(notably, high content of Asparagine(D), Glycine(G) and Phenylamine(F), with balanced leucine(L) and methionine(M) percent composition of 7.7%; and large negative(-6) charged. The latter, charge orientation and amino acid content, have lately been determined to be the major determinants for interaction between acidic termini of TcP0 and TcP2β and specific auto- antibodies(**see** **Figure.1** **for illustration**) [17]. Our search for alternate such possible related chemical species, nevertheless, failed to yield any matching chemical species. There is hence need to conduct metabolome wide association studies (MWAS) to ascertain what metabolites are common among persons with EMF or EMF preceded hypereosinophilia.

We note a number of specific short -comings of using the BLAST-P method for this study. **First**, while it is clear that protein and peptide composition are important for their biological function, the same is not always straightforward in their primary structure formats. Rather, the 3D structure and the dynamics of peptides and proteins in solution are much more important, in terms of biological function. **Against this background, perhaps this study would better have been conducted using 3-D structural searches**. All in all, it is widely established that only those proteins with a sequence identity of at least ∼30% are highly probable to share an evolutionary ancestor and to share the same overall fold (with the probability increasing as the identity sequence increase)[26], [28]. **Second**, in as much as the afore-going account is a useful rule of thumb for proteins in general, it is likely that, for peptides, the panorama is much more complex. For instance, as described by Martin et *al*. [17], the C-terminal portion of P2B protein from Leishmania *brazilensis*, Trypanosoma *cruzi* and Homo *sapiens*, respectively differ in only one amino acid. However, while R13 and H13 are capable of binding to a specific monoclonal antibody; A13 is not capable of such action. On the other hand, the second extracellular loop of rhodopsine is able to bind to the same antibody (less tightly) in spite of the poor sequence similarity to R13 or H13. Lastly, even with our positive findings, there is a slight possibility that no shared mechanism of pathology in Davies and Chaga's diseases actually exists. In assuming the latter position (one that is likely for some as our study coins no particular EMF causative insult), those of this line of philosophy may need to note that it is equally agreeable at the moment to further postulate that the fundamental mechanisms of aetiology are similar (in that, they involve autoimmunity), but EMF responsible auto-antigens are completely different from the acidic termini of the T.*cruzi* ribosomal proteins TcP0 and TcP2β that cause disease in Chaga's [16], [17]. Again, such differences, we sustain can only but be speculative; given that our search for related chemical species in known databases by way of NIST and SureChem yielded no currently known species with the primary structures C_61_H_83_N_13_O_26_S_1_and C_64_H_87_N_13_O_28_S_1_ consistent with the T.*cruzi* ribosomal acidic C-termini peptides. This also rules-out the possibility that previous chemical toxins such as Cerium, Thorium [5], and Arsenic [9] may mediate EMF through similar antigens/charge as do the acidic termini of TcP0 and TcP2β, for Chaga's diseases.

Lastly, besides the above noted potential shortcomings of our methodologies; two outstanding ambiguities in our study findings warrant further discussion and explanation. **First**, that the NCBI BLAST-P tool at default setting yielded results contradictory of existing data on the phylogenetic relationship of ribosomal P protein C-terminal repeats among eukaryotes [26]–[29] requires explanation. Need for such an explanation was, objectively, made further necessary in light of the fact that- in a recent study, we successfully used related approaches to evidence the species specific conservation of a sub-group of mosquito non- Long Terminal Repeat (non-LTR) small coding RNAs of the Long Interspersed Nuclear Elements (LINE) class- retroposons [32]; with insignificant differences observed from results obtained by other tools including a protein clock and genome cross-referencing or XREFdb [33]–[35]. However, in order to reduce errors in alignment searches, the default setting of the NCBI BLAST-P tool are designed not to permit database searches employing short-repetitive sequence queries [18], [19]. Therefore, unless the filter is turned off, no result (hit) can be obtained-the case observed in our report. **Second**, despite both peptides sharing related negative charge orientation, closely similar structures and amino acids content (**see** **Figure 1**), their respective predicted Instability Indices (II) were differing, with TcP0 being stable (at an II value of 11.7) while TcP2β is unstable (II value of 83.69). Generally, any peptide with an II above 40 is denoted as unstable [25]. This was interpreted-likely prematurely; as indicative of the possibility that variability in stability of both peptides per se does not influence their biological half life, both of which are shown to be 1 hour within mammalian reticulocytes (see Supplementary files 2 and 3). **Third and last**, while previous studies of EMF clearly show the possible role of genetic variants, geographical locales and socioeconomic status in the etiology of EMF [5], [6], our work is limited in that, although studying the insults possibly common in these groups, it never took consideration of those other factors including age and genetic variations. Specifically, Freer et *al.* [6], in an unmatched case control study in Mulago Hospital, Kampala of 61 EMF patients and 120 controls, show that EMF patients were significantly more likely than controls to have Rwanda/Burundi ethnic origins (P = 0.008), be peasants (P<0.001), and to come from defined geographical locations (P = 0.003). Elsewhere, Mocumbi *et al.* [11] not only highlighted the familial and endemic nature of the disease in tropics but also identified early disease and asymptomatology to occur among such subjects. Therefore, amidst the emerging role of genomics in infectious and neglected tropical diseases [36], it may equally be necessary to conduct genome wide association studies (GWAS) to establish the particular small nuclear polymorphisms (SNPs) among persons from these established ethnic and geographical locales that make them highly susceptible to EMF. The fusion protein FIP1L1-PDGFRa, a constitutively activated tyrosine kinase found in as many as half of those with the idiopathic hypereosinophilic syndrome, has emerged as a therapeutic target for imatinib [5], [37]. The recent finding that serotonin acts as a chemotactic factor for eosinophils also underlines the need for inquiries into the role of this pathway in EMF [38]. Zanettini and colleagues [39] have found that some anti- Parkinson medications induce valvular fibrosis via their action on 5HT_2B_ receptors. GWAS studies are called for to determine whether or not, polymorphisms in these and other receptors influence susceptibility to EMF in the presence of intermittent Eosinophilia, in which case, existing drugs may be tried in EMF.

In conclusion, our findings provide a “window” to suggest that cross reactivity of antibodies against ribosomal P protein C-termini of several animal, plant and protozoal with heart tissue may mediate EMF in a similar manner as C- termini of T. *cruzi* do for Chaga's disease. It is, however, equally possible that the mechanisms of molecular mimicry between the suspected EMF-insults and myocardial tissue are mediated via different myocardial antigens- thereby, denoting these-our study alluded species-protein portions, not the likely cause of EMF.

## Materials and Methods

### A. Comparative alignment of TcP0 and TcP2β acidic terminal Sequences with over 1, 789 organismal Proteomes

#### A1. NCBI BLAST-P

Sequence alignments were conducted by querying *sequences of the acidic termini* of the two T.*cruzi* ribosomal P proteins TcP0 and TcP2β [ the latters' Swiss Prot Accessions numbers are P26796 and Q26957, respectively]'s known to mediate autoimmune responses in Chaga's diseases: EDDDDDFGMGALF and EEEDDDMGFGLFD across an over 1,789 organismal proteome database, 20 plant (including cassava) proteome database by way of BLAST-P Software and Algorithms[18], [19] at default setting[ and hence after, repeated with altered algorithms E = 1–10, A & D = 10–100]. On the other hand, similarity to viral genomes was determined by querying entire peptides' cross referenced DNA/mRNA sequences (derived from mRNA sequences of mRNA of the TcP0 [20], [21], [22] -NCBI Accession X65066; and the whole genomic short gun of T.*cruzi* [23], [24]- NCBI Accession NW_001849569) across a database of ssRNA, ssDNA, dsRNA and dsDNA viral genomes employing NCBI's viral genotyping and dsDNA BLAST-N respectively[18], [19]. The latter approach( use of whole reference gene nucleotide sequences rather than amino acids sequences] was employed because the currently available BLAST algorithm and software linked to the viral databases only support nucleotide (N) searches

#### A.2 SIB BLAST-P Tool

**(**available at URL**:** http://www.expasy.ch/cgi-bin/blast.pl)

Repeat alignment of acidic termini of TcP0 and TcP2β with sequences of proteins available in the Swiss Prot database using BLAST-P at SIB were conducted as prior described by Altschul et *al.*, [18], [19].

### B. Computational Derivation of TcP0 and TcP2β acidic terminal chemical composition

To be able to search across chemical species databases (see method 3 below), we needed to derive the chemical formulae of the acidic termini of both TcP0 and TcP2β. The Expasy software Protparam [25] was used to achieve this. Briefly, Protparam [25] is a proprietary computational software available at Swiss Prot/UniProt that can be used to derive series biophysical profiles as well as determine the amino acid composition of any protein or polypeptide of interest, provided its primary structure (linear alignment of amino acids) is known. The Software has a user interface that allows one to feed the respective peptide or polypeptide primary structure into it, hence fore computing the parameters.

### C. Searching for homologous chemical species to TcP0 and TcP2β acidic termini

The chemical formulae of the acidic termini of TcP0 and TcP2β obtained above: C_61_H_83_N_13_O_26_S_1_and C_64_H_87_N_13_O_28_S_1_ respectively, were used to search for relationship to chemical toxins like Cerium, Thorium, Arsenic, Serotonin, or Vitamin D that are suspected causative insults for EMF[5] as well as all other known chemical species. The National Institute of Standard and Technology(NIST) and SureChem Chemical searches(for details, see URL link below) were finally employed to relate the former formulae to chemical formulae and structure of all known compounds in the respective databases.

### D. Databases, Software and Algorithms

The Databases, Software and algorithms used in this study can all freely be accessed by the reader at the following world-wide web sites:

- All NCBI BLAST Assembled Organismal genomes are available at the NCBI URL:http://www.ncbi.nlm.nih.gov/mapview/
- *SIB BLAST Tool is available at the following Expasy URL*: http://www.expasy.ch/cgi-bin/blast.pl)
- The NCBI Viral BLAST against dsDNA viruses is available at the NCBI URL:http://www.ncbi.nlm.nih.gov/genomes/VIRUSES/Bitor.cgi?db=VOG&data=vog&gdata=dsdna.defl
- The NCBI Viral Genotyping BLAST tool is available at the NCBI URL:http://www.ncbi.nlm.nih.gov/projects/genotyping/formpage.cgi
- The Expasy Software Protparam used to determine chemical composition is available at the Swiss Prot/Uniprot URL: http://www.expasy.ch/tools/protparam.html
- The SureChem software and algorithm is available at the URL: http://www.freepatentsonline.com/
- The National Institute of Standards and Technology(NIST) Chemical Formula Search Tool is available at the NIST URL: http://webbook.nist.gov/chemistry/form-ser.html

## Supporting Information

Figure S1 Showing the Taxonomic Tree of all BLAST hits obtained by querying the C-termini of TcP2β across a Swiss Prot database using the BLAST tool at SIB. The Figure illustrates the Taxonomic relationship, rooted on the query, of all hits obtained by querying the 13 amino acid sequences of the C-termini of TcP2β (EEEDDDMGFGLFD) across a Swiss Prot database using the BLAST tool at SIB. The data was generated by the BLAST tool at the following URL: SIB availablehttp://www.expasy.ch/cgi-bin/blast.pl
(0.03 MB PDF)

File S1 Showing the only hits to the source of the two C-terminal peptides of the ribosomal proteins TcP0: the 60S acidic ribosomal protein P0 of T.cruzi using the BLAST-P tool at NCBI( Default settings). This file illustrates the E-values and score, description and details of the hits obtained by querying the acidic termini of the T.cruzi ribosomal proteins TcP0 against a genome wide database of 31 protozoa proteins. Note that despite the presence in the database of pathogens previously suspected to be the causative insult of EMF such as plasmodia [5], the only hits were those to the source of the query peptide TcP0: the 60S acidic ribosomal protein P0 of T.cruzi. The data was generated by the NCI BLAST-P Software and algorithms [18], [19]. Similar Data obtained with the C-terminus of TcP2β is not shown. These results are explained by the fact that in order to reduce errors in alignment searches, the default settings do not permit database searches that employ short-repetitive queries [18], [19]. Therefore, unless the filter is turned off, no results will be found.
(0.07 MB DOC)

File S2 Showing Scores and E values of hits obtained querying C-termini of TcP2β across a Swiss Prot database using the BLAST tool at SIB. Showing Scores and E values of hits obtained by querying the 13 amino acid sequences of the C-termini of TcP2β (EEEDDDMGFGLFD) across a Swiss Prot database using the BLAST tool at SIB. The data was generated by the BLAST tool at the following URL: SIB availablehttp://www.expasy.ch/cgi-bin/blast.pl
(0.87 MB DOC)

File S3 Showing the Biophysical profiles of the acidic C-terminus of the T.cruzi ribosomal P protein TcP0. The file provides details of the biophysical profiles, including chemical structure, amino acid composition, Theoretical PI, Instability index, Extinction coefficient Aliphatic Index and Graavy of the C-terminus peptide of TcP0 (EDDDDDFGMGALF). The data was generated by the Expasy software Protparam [25]
(0.24 MB DOC)

File S4 Showing the Biophysical profiles of the acidic C-terminus of the T.cruzi ribosomal P protein TcP2β. The file provides details of the biophysical profiles, including chemical structure, amino acid composition, Theoretical PI, Instability index, Extinction coefficient Aliphatic Index and Graavy of the C-terminus peptides of TcP2β (EEEDDDMGFGLFD). The data was generated by the Expasy software Protparam [25]
(0.24 MB DOC)

## References

[1] Parry EH, Abrahams DG (1965). The natural history of endomyocardial fibrosis.. Q J Med.

[2] Connor DH, Somers K, Hutt MS, Manion WC, D'Arbela PG (1967). Endomyocardial fibrosis in Uganda (Davies' disease): An epidemiologic, clinical, and pathologic study.. Am Heart J.

[3] Davies JNP (1948). Endomyocardial fibrosis in Uganda.. East Afr Med J.

[4] Sivasankaran S (2009). Restrictive cardiomyopathy in India: the story of a vanishing mystery.. Heart.

[5] Bukhman G, Ziegler J, Parry E (2008). Endomyocardial Fibrosis: Still a Mystery after 60 Years.. PLoS Negl Trop.

[6] Freers J, Mayanja-Kizza H, Rutakingirwa M, Gerwing E (1996). Endomyocardial fibrosis: why is there striking ascites with little or no peripheral oedema?. Lancet.

[7] Andy JJ, Ogunowo PO, Akpan NA, Odigwe CO, Ekanem IA (1998). Helminth associated hypereosinophilia and tropical endomyocardial fibrosis (EMF) in Nigeria.. Acta Trop.

[8] Beisel WR (1995). Herman award lecture, 1995: infection-induced malnutrition - from cholera to cytokines.. Am J Clin Nutr.

[9] Edge J (1946). Myocardial fibrosis following arsenical therapy: report of a case.. Lancet.

[10] Iglezias SD, Benvenuti LA, Calabrese F, Salemi VM, Silva AM (2008). Endomyocardial fibrosis: pathological and molecular findings of surgically resected ventricular endomyocardium.. Virchows Arch.

[11] Mocumbi AO, Ferriera MB, Sidi D (2008). A population study of endomycardial fibrosis in a rural area of Mozambique.. N Engl J Med.

[12] Rutakingirwa M, Ziegler JL, Newton R, Freers J (1999). Poverty and eosinophilia are risk factors for endomyocardial fibrosis (EMF) in Uganda.. Trop Med Int Health.

[13] Spry CJ, Take M, Tai PC (1985). Eosinophilic disorders affecting the myocardium and endocardium: a review.. Heart Vessels.

[14] Ntusi NB, Mayosi BM (2009). Epidemiology of heart failure in sub-Saharan Africa.. Expert Rev Cardiovasc Ther.

[15] Turan AA, Karayel F, Akyildiz EU, Ozdes T, Yilmaz E (2008). Sudden death due to eosinophilic endomyocardial diseases: three case reports.. Am J Forensic Med Pathol.

[16] Sepulveda P, Liegeard P, Wallukat G, Levin MJ, Hontebeyrie M (2000). Modulation of Cardiocyte Functional Activity by Antibodies against *Trypanosoma cruzi* Ribosomal P2 Protein C Terminus.. Infection and Immunity.

[17] Martin OA, Villegas ME, Aguilar CF (2009). Three-dimensional studies of pathogenic peptides from the c-terminal of Trypanosoma cruzi ribosomal P proteins and their interaction with a monoclonal antibody structural model.. PMC Biophys.

[18] Altschul SF, Madden TL, Schäffer AA, Zhang J, Zhang Z (1997). Gapped BLAST and PSI-BLAST: a new generation of protein database search programs.. Nucleic Acids Res.

[19] Altschul SF, Wootton JC, Gertz EM, Agarwala R, Morgulis A (2005). Protein database searches using compositionally adjusted substitution matrices.. FEBS J.

[20] Skeiky YA, Benson DR, Parsons M, Elkon KB, Reed SG (1992). Cloning and expression of Trypanosoma cruzi ribosomal protein P0 and epitope analysis of anti-P0 autoantibodies in Chagas' disease patients.. J Exp Med.

[21] Schijman AG, Levin MJ (1992). Nucleotide sequence of a cDNA encoding a Trypanosoma cruzi acidic ribosomal PO protein: a novel C-terminal domain in T. cruzi ribosomal P proteins.. Nucleic Acids Res.

[22] Schijman AG, Levitus G, Levin MJ (1992). Characterization of the C-terminal region of a Trypanosoma cruzi 38-kDa ribosomal P0 protein that does not react with lupus anti-P autoantibodies.. Immunol Lett.

[23] Schijman AG, Dusetti NJ, Vazquez MP, Lafon S, Levy-Yeyati P (1990). Nucleotide cDNA and complete deduced amino acid sequence of a Trypanosoma cruzi ribosomal P protein (P-JL5).. Nucleic Acids Res.

[24] El-Sayed NM, Myler PJ, Bartholomeu DC, Nilsson D, Aggarwal G (2005). The genome sequence of Trypanosoma cruzi, etiologic agent of Chagas disease.. Science.

[25] Gasteiger E, Hoogland C, Gattiker, Duvaud S, Wilkins MR, Walker JM (2005). Protein Identification and Analysis Tools on the ExPASy Server.. The Proteomics Protocols Handbook.

[26] Grela P, Bernadó P, Svergun D, Kwiatowski J, Abramczyk D (2008). Structural Relationships Among the Ribosomal Stalk Proteins from the Three Domains of Life.. J Mol Evol.

[27] Hasler P, Brot N, Weissbach H, Parnassa AP, Elkon KB (1991). Ribosomal proteins P0, P1, and P2 are phosphorylated by casein kinase II at their conserved carboxyl termini.. J Biol Chem.

[28] Gagou ME, Gabriel MAR, Ballesta JPG, Kouyanou S (2000). The ribosomal P-proteins of the medfly *Ceratitis capitata* form a heterogeneous stalk structure interacting with the endogenous P-proteins, in conditional P0-null strains of the yeast *Saccharomyces cerevisiae*.. Nucl Acids Res.

[29] Lewis CE (1958). Timed excretion of 5-hydroxy indoleacetic acid after oral administration of bananas and 5-hydroxytryptamine.. Proc Soc Exp Biol Med.

[30] Crawford MA (1962). Excretion of 5-hydroxyindolylacetic acid in East Africans.. Lancet.

[31] Ojo GO (1970). The pathogenesis of endomyocardial fibrosis: the question of 5-hydroxytryptamine.. Br Heart J.

[32] Byarugaba W, Kajumbula H, Wayengera M (2009). *In Silico* evidence for the species-specific conservation of mosquito retroposons: implications as a molecular biomarker.. Theor Biol Med Model.

[33] Feng DF, Cho G, Doolittle RF (1997). Determining divergence times with a protein clock: Update and reevaluation.. Proc Natl Acad Sci USA.

[34] Bassett DE, Boguski MS, Spencer F, Reeves R, Kim S (1997). Genome cross-referencing and XREFdb: Implications for the identification and analysis of genes mutated in human disease.. Nature Genet.

[35] Osamu G (2008). A space-efficient and accurate method for mapping and aligning cDNA sequences onto genomic sequence.. Nucl Acids Res.

[36] Wayengera M, Byarugaba W (2008). Emphasizing the vitality of genomics related research in the area of Infectious diseases.. Sci Res Essay.

[37] Cools J, DeAngelo DJ, Gotlib J, Stover EH, Legare RD (2003). A tyrosine kinase created by fusion of the PDGFRA and FIP1L1 genes as a therapeutic target of imatinib in idiopathic hypereosinophilic syndrome.. N Engl J Med.

[38] Boehme SA, Lio FM, Sikora L, Pandit TS, Lavrador K (2004). Cutting edge: serotonin is a chemotactic factor for eosinophils and functions additively with eotaxin.. J Immunol.

[39] Zanettini R, Antonini A, Gatto G, Gentile R, Tesei S (2007). Valvular heart disease and the use of dopamine agonists for Parkinson's disease.. N Engl J Med.

