# Supplementary material for: Searching for New Clues about the Molecular Cause of Endomyocardial Fibrosis by Way of In Silico Proteomics and Analytical Chemistry
Source: PLoS One. 2009 Oct 12;4(10):e7420. doi: 10.1371/journal.pone.0007420 (PMC2757908; doi:10.1371/journal.pone.0007420)
Supplement: Figure S1 — Showing the Taxonomic Tree of all BLAST hits obtained by querying the C-termini of TcP2β across a Swiss Prot database using the BLAST tool at SIB. The Figure illustrates the Taxonomic relationship, rooted on the query, of all hits obtained by querying the 13 amino acid sequences of the C-termini of TcP2β (EEEDDDMGFGLFD) across a Swiss Prot database using the BLAST tool at SIB. The data was generated by the BLAST tool at the following URL: SIB availablehttp://www.expasy.ch/cgi-bin/blast.pl (0.03 MB PDF) [file pone.0007420.s001.pdf]

Taxonomic view of SIB BLAST hits

root - 100

Eukaryota - 100

Alveolata - 1

Babesia rodhaini - 1

Euglenozoa - 36

Trypanosomatidae - 36

Leishmania - 18

Leishmania braziliensis species complex - 6

Leishmania braziliensis - 5

Leishmania peruviana - 1

Leishmania donovani - 3

Leishmania infantum - 5

Leishmania major - 4

Trypanosoma - 18

Schizotrypanum - 13

Trypanosoma cruzi - 13

Trypanosoma brucei - 4

Trypanosoma brucei brucei - 1

Fungi - 8

Ascomycota - 5

Saccharomycetaceae - 5

Eremothecium - 1

Ashbya gossypii (Yeast) (Eremothecium gossypii) - 1

Kluyveromyces - 1

Kluyveromyces lactis (Yeast) (Candida sphaerica) - 1

Vanderwaltozyma - 3

Vanderwaltozyma polyspora (strain ATCC 22028 / DSM 70294) (Kluyveromyces polysporus) - 3

Basidiomycota - 1

Melampsora medusae f. sp. deltoidis - 1

Dikarya - 2

Saccharomycetaceae - 2

Pichia - 1

Pichia pastoris (strain GS115) (Yeast) - 1

Saccharomyces - 1

Saccharomyces cerevisiae (Baker's yeast) - 1

Metazoa - 55

Annelida - 1

Pectinaria gouldii (Trumpet worm) (Ice-cream cone worm) - 1

Arthropoda - 33

Chelicerata - 6

Acari - 6

Acariformes - 1

Blomia tropicalis (Mite) - 1

Parasitiformes - 5

Ixodoidea - 5

Argasidae - 1

Argas monolakensis (Mono lake bird tick) - 1

Ixodidae - 4

Haemaphysalinae - 2

Haemaphysalis - 2

Haemaphysalis longicornis (Bush tick) - 1

Haemaphysalis qinghaiensis - 1

Ixodinae - 2

Ixodes scapularis (Black-legged tick) (Deer tick) - 2

Crustacea - 5

Branchiopoda - 3

Artemia - 3

Artemia salina (Brine shrimp) - 2

Artemia sanfranciscana (Brine shrimp) (Artemia franciscana) - 1

Maxillopoda - 2

Lepeophtheirus salmonis (salmon louse) - 2

Hexapoda - 22

Neoptera - 22

Endopterygota - 15

Diptera - 13

Muscomorpha - 13

Ephydroidea - 11

Drosophila - 11

Drosophila ananassae (Fruit fly) - 1

Drosophila erecta (Fruit fly) - 1

Drosophila grimshawi(Fruit fly)(Idiomyia grimshawi-1

Drosophila mojavensis (Fruit fly) - 1

Drosophila pseudoobscura pseudoobscura (Fruit fly) - 1

Drosophila sechellia (Fruit fly) - 1

Drosophila simulans (Fruit fly) - 1

Drosophila virilis (Fruit fly) - 1

Drosophila willistoni (Fruit fly) - 1

Drosophila yakuba (Fruit fly) - 1

Sophophora - 1

Drosophila melanogaster (Fruit fly) - 1

Oestroidea - 1

Sarcophaga crassipalpis - 1

Tephritoidea - 1

Ceratitis capitata (Mediterranean fruit fly) - 1

Lepidoptera - 2

Bombyx mori (Silk moth) - 2

Paraneoptera - 7

Sternorrhyncha - 7

Aphidiformes - 6

Aphidoidea - 5

Aphididae - 5

Aphidini - 1

Toxoptera citricida (Brown citrus aphid) - 1

Macrosiphini - 4

Acyrthosiphon pisum (Pea aphid) - 4

Coccoidea - 1

Maconellicoccus hirsutus (Pink hibiscus mealybug)- 1

Psylliformes - 1

Diaphorina citri (Asian citrus psyllid)- 1

Chordata - 5

Branchiostoma - 5

Branchiostoma belcheri (Amphioxus) - 1

Branchiostoma floridae (Florida lancelet) (Amphioxus) - 4

Cnidaria - 4

Hexacorallia - 4

Actiniaria - 3

Nematostella vectensis (Starlet sea anemone) - 3

Scleractinia - 1

Stylophora pistillata - 1

Echinodermata - 1

Asterina pectinifera (Starfish)(Patiria pectinifera) - 1

Mollusca - 2

Bivalvia - 1

Mytilus galloprovincialis (Mediterranean mussel) - 1

Polyplacophora - 1

Cryptochiton stelleri - 1

Nemertea - 3

Lineus viridis - 3

Placozoa - 3

Trichoplax adhaerens - 3

Porifera - 3

Suberites domuncula (Sponge) - 3
