# Supplementary material for: Searching for New Clues about the Molecular Cause of Endomyocardial Fibrosis by Way of In Silico Proteomics and Analytical Chemistry
Source: PLoS One. 2009 Oct 12;4(10):e7420. doi: 10.1371/journal.pone.0007420 (PMC2757908; doi:10.1371/journal.pone.0007420)
Supplement: File S1 — Showing the only hits to the source of the two C-terminal peptides of the ribosomal proteins TcP0: the 60S acidic ribosomal protein P0 of T.cruzi using the BLAST-P tool at NCBI( Default settings). This file illustrates the E-values and score, description and details of the hits obtained by querying the acidic termini of the T.cruzi ribosomal proteins TcP0 against a genome wide database of 31 protozoa proteins. Note that despite the presence in the database of pathogens previously suspected to be the causative insult of EMF such as plasmodia [5], the only hits were those to the source of the query peptide TcP0: the 60S acidic ribosomal protein P0 of T.cruzi. The data was generated by the NCI BLAST-P Software and algorithms [18], [19]. Similar Data obtained with the C-terminus of TcP2β is not shown. These results are explained by the fact that in order to reduce errors in alignment searches, the default settings do not permit database searches that employ short-repetitive queries [18], [19]. Therefore, unless the filter is turned off, no results will be found. (0.07 MB DOC) [file pone.0007420.s002.doc]

1. **Search Definition(s)**

Query ID :lcl|8023

Description : None

Molecule type : amino acid

Query Length :13

Database Name: 31 databases

| **Database** | **Description** | **Posted Date** |
| --- | --- | --- |
| Microbial/5865 | Unfinished Babesia bovis proteins | Jun 24, 2009 5:41 PM |
| Microbial/484906 | Completed Babesia bovis T2Bo proteins | Jun 24, 2009 5:41 PM |
| Microbial/237895 | Unfinished Cryptosporidium hominis proteins | Jun 24, 2009 5:41 PM |
| Microbial/353152 | Cryptosporidium parvum Iowa II proteins | Jun 24, 2009 5:41 PM |
| Microbial/413949 | Completed Eimeria tenella str. Houghton proteins | Jun 24, 2009 5:41 PM |
| Microbial/5821 | Plasmodium berghei proteins | Jun 24, 2009 5:41 PM |
| Microbial/31271 | Plasmodium chabaudi chabaudi proteins | Jun 24, 2009 5:41 PM |
| Microbial/36329 | Completed Plasmodium falciparum 3D7 proteins | Jun 24, 2009 5:41 PM |
| Microbial/5851 | Completed Plasmodium knowlesi strain H proteins | Jun 24, 2009 5:41 PM |
| Microbial/5855 | Unfinished Plasmodium vivax proteins | Jun 24, 2009 5:41 PM |
| Microbial/126793 | Plasmodium vivax SaI-1 proteins | Jun 24, 2009 5:41 PM |
| Microbial/73239 | Plasmodium yoelii yoelii proteins | Jun 24, 2009 5:41 PM |
| Microbial/353154 | Completed Theileria annulata strain Ankara proteins | Jun 24, 2009 5:41 PM |
| Microbial/5875 | Unfinished Theileria parva proteins | Jun 24, 2009 5:41 PM |
| Microbial/333668 | Completed Theileria parva strain Muguga proteins | Jun 24, 2009 5:41 PM |
| Microbial/420245 | Completed Leishmania braziliensis MHOM/BR/75/M2904 proteins | Jun 24, 2009 5:41 PM |
| Microbial/435258 | Completed Leishmania infantum JPCM5 proteins | Jun 24, 2009 5:41 PM |
| Microbial/347515 | Completed Leishmania major strain Friedlin proteins | Jun 24, 2009 5:41 PM |
| Microbial/185431 | Completed Trypanosoma brucei TREU927 proteins | Jun 24, 2009 5:41 PM |
| Microbial/5693 | Unfinished Trypanosoma cruzi proteins | Jun 24, 2009 5:41 PM |
| Microbial/353153 | Unfinished Trypanosoma cruzi strain CL Brener proteins | Jun 24, 2009 5:41 PM |
| Microbial/227086 | Completed Bigelowiella natans proteins | Jun 24, 2009 5:41 PM |
| Microbial/2762 | Completed Cyanophora paradoxa proteins | Jun 24, 2009 5:41 PM |
| Microbial/352472 | Dictyostelium discoideum AX4 proteins | Jun 24, 2009 5:41 PM |
| Microbial/184922 | Giardia lamblia ATCC 50803 proteins | Jun 24, 2009 5:41 PM |
| Microbial/55529 | Completed Guillardia theta proteins | Jun 24, 2009 5:41 PM |
| Microbial/464988 | Completed Hemiselmis andersenii proteins | Jun 24, 2009 5:41 PM |
| Microbial/431895 | Completed Monosiga brevicollis MX1 proteins | Jun 24, 2009 5:41 PM |
| Microbial/556484 | Completed Phaeodactylum tricornutum CCAP 1055/1 proteins | Jun 24, 2009 5:41 PM |
| Microbial/312017 | Completed Tetrahymena thermophila SB210 proteins | Jun 24, 2009 5:41 PM |
| Microbial/296543 | Completed Thalassiosira pseudonana CCMP1335 proteins | Jun 24, 2009 5:41 PM |

B) Description

Score E

Sequences producing significant alignments: (Bits) Value

[gb|EAN99267.1|](http://www.ncbi.nlm.nih.gov/entrez/query.fcgi?cmd=Retrieve&db=Protein&list_uids=70886487&dopt=GenPept&RID=47KNE89H013&log$=prottop&blast_rank=1) 60S acidic ribosomal protein P0 [Trypanosoma c... [29.3](http://www.ncbi.nlm.nih.gov/blast/Blast.cgi#70886487%2370886487) 2.2 [
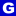
](http://www.ncbi.nlm.nih.gov/entrez/query.fcgi?db=gene&cmd=search&term=70886487%5BPUID%5D&RID=47KNE89H013&log$=genetop&blast_rank=1)

[gb|EAN99266.1|](http://www.ncbi.nlm.nih.gov/entrez/query.fcgi?cmd=Retrieve&db=Protein&list_uids=70886486&dopt=GenPept&RID=47KNE89H013&log$=prottop&blast_rank=2) 60S acidic ribosomal protein P0 [Trypanosoma c... [29.3](http://www.ncbi.nlm.nih.gov/blast/Blast.cgi#70886486%2370886486) 2.2 [
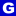
](http://www.ncbi.nlm.nih.gov/entrez/query.fcgi?db=gene&cmd=search&term=70886486%5BPUID%5D&RID=47KNE89H013&log$=genetop&blast_rank=2)

**c) Details**

>[gb|EAN99267.1|](http://www.ncbi.nlm.nih.gov/entrez/query.fcgi?cmd=Retrieve&db=Protein&list_uids=70886487&dopt=GenPept&RID=47KNE89H013&log$=protalign&blast_rank=1) [
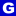
](http://www.ncbi.nlm.nih.gov/entrez/query.fcgi?db=gene&cmd=search&term=70886487%5BPUID%5D&RID=47KNE89H013&log$=genealign&blast_rank=1) 60S acidic ribosomal protein P0 [Trypanosoma cruzi]

Length=323

[GENE ID: 3553968 Tc00.1047053508355.260](http://www.ncbi.nlm.nih.gov/sites/entrez?db=gene&cmd=search&term=3553968&RID=47KNE89H013&log$=geneexplicitprot&blast_rank=1) | 60S acidic ribosomal protein P0

[Trypanosoma cruzi strain CL Brener]

Score = 29.3 bits (64), Expect = 2.2, Method: Compositional matrix adjust.

Identities = 13/13 (100%), Positives = 13/13 (100%), Gaps = 0/13 (0%)

Query 1 EDDDDDFGMGALF 13

EDDDDDFGMGALF

Sbjct 311 EDDDDDFGMGALF 323

>[gb|EAN99266.1|](http://www.ncbi.nlm.nih.gov/entrez/query.fcgi?cmd=Retrieve&db=Protein&list_uids=70886486&dopt=GenPept&RID=47KNE89H013&log$=protalign&blast_rank=2) [
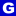
](http://www.ncbi.nlm.nih.gov/entrez/query.fcgi?db=gene&cmd=search&term=70886486%5BPUID%5D&RID=47KNE89H013&log$=genealign&blast_rank=2) 60S acidic ribosomal protein P0 [Trypanosoma cruzi]

Length=323

[GENE ID: 3553967 Tc00.1047053508355.250](http://www.ncbi.nlm.nih.gov/sites/entrez?db=gene&cmd=search&term=3553967&RID=47KNE89H013&log$=geneexplicitprot&blast_rank=2) | 60S acidic ribosomal protein P0

[Trypanosoma cruzi strain CL Brener]

Score = 29.3 bits (64), Expect = 2.2, Method: Compositional matrix adjust.

Identities = 13/13 (100%), Positives = 13/13 (100%), Gaps = 0/13 (0%)

Query 1 EDDDDDFGMGALF 13

EDDDDDFGMGALF

Sbjct 311 EDDDDDFGMGALF 323
