# Supplementary material for: Searching for New Clues about the Molecular Cause of Endomyocardial Fibrosis by Way of In Silico Proteomics and Analytical Chemistry
Source: PLoS One. 2009 Oct 12;4(10):e7420. doi: 10.1371/journal.pone.0007420 (PMC2757908; doi:10.1371/journal.pone.0007420)
Supplement: File S2 — Showing Scores and E values of hits obtained querying C-termini of TcP2β across a Swiss Prot database using the BLAST tool at SIB. Showing Scores and E values of hits obtained by querying the 13 amino acid sequences of the C-termini of TcP2β (EEEDDDMGFGLFD) across a Swiss Prot database using the BLAST tool at SIB. The data was generated by the BLAST tool at the following URL: SIB availablehttp://www.expasy.ch/cgi-bin/blast.pl (0.87 MB DOC) [file pone.0007420.s003.doc]

| [**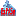ExPASy Home page**](http://www.expasy.ch/) | [**Site Map**](http://www.expasy.ch/sitemap.html) | [**Search ExPASy**](http://www.expasy.ch/ExpasyHunt/) | [**Contact us**](http://www.expasy.ch/contact.html) | [**Swiss-Prot**](http://www.expasy.ch/sprot/) | [**Proteomics tools**](http://www.expasy.ch/tools/) |
| --- | --- | --- | --- | --- | --- |

If results of this search are reported or published, please mention that the computation was performed at the SIB using the BLAST network service.

NCBI BLAST program reference [PMID:[9254694](http://www.ncbi.nlm.nih.gov/entrez/query.fcgi?cmd=Retrieve&db=PubMed&list_uids=9254694&dopt=Abstract)]:

Altschul S.F., Madden T.L., Schäffer A.A., Zhang J., Zhang Z., Miller W.,Lipman D.J. Gapped BLAST and PSI-BLAST: a new generation of protein database search programs. Nucleic Acids Res. 25:3389-3402(1997).

============================================================================

Query: 13 AA EEEDDDMGFGLFD

Date run: 2009-09-11 09:01:11 UTC+0100 on blast01.vital-it.ch

Program: NCBI BLASTP 2.2.17 [Aug-26-2007]

Database: UniProtKB 9,670,896 sequences; 3,150,505,678 letters

**List of potentially matching sequences**

Top of Form

Include query sequence


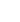
 Db AC Description Score E-value

sp [P26795](http://www.uniprot.org/uniprot/P26795) RLA3_TRYCR 60S acidic ribosomal protein P2-B (P2B) [Tr... [47](http://www.expasy.ch/cgi-bin/blast.pl#A1%23A1) 2e-04

sp [P23632](http://www.uniprot.org/uniprot/P23632) RLA2_TRYCR 60S acidic ribosomal protein P2-A (P) (P-JL... [47](http://www.expasy.ch/cgi-bin/blast.pl#A2%23A2) 2e-04

sp [P26643](http://www.uniprot.org/uniprot/P26643) RLA1_TRYCR 60S acidic ribosomal protein P1 [Trypanosom... [47](http://www.expasy.ch/cgi-bin/blast.pl#A3%23A3) 2e-04

sp [P19889](http://www.uniprot.org/uniprot/P19889) RLA0_DROME 60S acidic ribosomal protein P0 (DNA-(apuri... [47](http://www.expasy.ch/cgi-bin/blast.pl#A4%23A4) 2e-04

tr [Q9NHP0](http://www.uniprot.org/uniprot/Q9NHP0) _SARCR 60S acidic ribosomal protein P0 [Sarcophaga cras... [47](http://www.expasy.ch/cgi-bin/blast.pl#A5%23A5) 2e-04

tr [Q6IWF5](http://www.uniprot.org/uniprot/Q6IWF5) _TRYCR Ribosomal P protein type 1 (60S acidic ribosomal... [47](http://www.expasy.ch/cgi-bin/blast.pl#A6%23A6) 2e-04

tr [Q5XUB6](http://www.uniprot.org/uniprot/Q5XUB6) _TOXCI Putative acidic p0 ribosomal protein [Toxoptera ... [47](http://www.expasy.ch/cgi-bin/blast.pl#A7%23A7) 2e-04

tr [Q4DWU6](http://www.uniprot.org/uniprot/Q4DWU6) _TRYCR 60S acidic ribosomal protein P2, putative [Tc00.... [47](http://www.expasy.ch/cgi-bin/blast.pl#A8%23A8) 2e-04

tr [Q4DLC3](http://www.uniprot.org/uniprot/Q4DLC3) _TRYCR 60S acidic ribosomal protein P2, putative [Tc00.... [47](http://www.expasy.ch/cgi-bin/blast.pl#A9%23A9) 2e-04

tr [Q4D991](http://www.uniprot.org/uniprot/Q4D991) _TRYCR 60S acidic ribosomal protein, putative [Tc00.104... [47](http://www.expasy.ch/cgi-bin/blast.pl#A10%23A10) 2e-04

tr [Q4D4B9](http://www.uniprot.org/uniprot/Q4D4B9) _TRYCR 60S acidic ribosomal protein P2, putative [Tc00.... [47](http://www.expasy.ch/cgi-bin/blast.pl#A11%23A11) 2e-04

tr [Q4D4B8](http://www.uniprot.org/uniprot/Q4D4B8) _TRYCR 60S acidic ribosomal protein P2, putative [Tc00.... [47](http://www.expasy.ch/cgi-bin/blast.pl#A12%23A12) 2e-04

tr [Q4CSS8](http://www.uniprot.org/uniprot/Q4CSS8) _TRYCR 60S acidic ribosomal protein P2, putative [Tc00.... [47](http://www.expasy.ch/cgi-bin/blast.pl#A13%23A13) 2e-04

tr [Q38EY6](http://www.uniprot.org/uniprot/Q38EY6) _9TRYP 60S acidic ribosomal protein, putative [Tb09.160... [47](http://www.expasy.ch/cgi-bin/blast.pl#A14%23A14) 2e-04

tr [Q29DM5](http://www.uniprot.org/uniprot/Q29DM5) _DROPS GA20389 [GA20389] [Drosophila pseudoobscura pseu... [47](http://www.expasy.ch/cgi-bin/blast.pl#A15%23A15) 2e-04

tr [Q27291](http://www.uniprot.org/uniprot/Q27291) _TRYCR TcP2beta protein [TcP2beta] [Trypanosoma cruzi] [47](http://www.expasy.ch/cgi-bin/blast.pl#A16%23A16) 2e-04

tr [Q26959](http://www.uniprot.org/uniprot/Q26959) _TRYCR TcP2beta protein [TcP2beta] [Trypanosoma cruzi] [47](http://www.expasy.ch/cgi-bin/blast.pl#A17%23A17) 2e-04

tr [Q26957](http://www.uniprot.org/uniprot/Q26957) _TRYCR 60S acidic ribosomal protein P2 beta (H6.4), put... [47](http://www.expasy.ch/cgi-bin/blast.pl#A18%23A18) 2e-04

tr [Q201W9](http://www.uniprot.org/uniprot/Q201W9) _ACYPI ACYPI000079 protein (Putative acidic p0 ribosoma... [47](http://www.expasy.ch/cgi-bin/blast.pl#A19%23A19) 2e-04

tr [Q0PWV6](http://www.uniprot.org/uniprot/Q0PWV6) _DIACI Putative acidic p0 ribosomal protein (Fragment) ... [47](http://www.expasy.ch/cgi-bin/blast.pl#A20%23A20) 2e-04

tr [Q09JS1](http://www.uniprot.org/uniprot/Q09JS1) _ARGMO Ribosomal protein LP1 [Argas monolakensis (Mono ... [47](http://www.expasy.ch/cgi-bin/blast.pl#A21%23A21) 2e-04

tr [C4WRM3](http://www.uniprot.org/uniprot/C4WRM3) _ACYPI ACYPI000079 protein [ACYPI000079] [Acyrthosiphon... [47](http://www.expasy.ch/cgi-bin/blast.pl#A22%23A22) 2e-04

tr [C4WRL7](http://www.uniprot.org/uniprot/C4WRL7) _ACYPI ACYPI000079 protein [ACYPI000079] [Acyrthosiphon... [47](http://www.expasy.ch/cgi-bin/blast.pl#A23%23A23) 2e-04

tr [B7PRG2](http://www.uniprot.org/uniprot/B7PRG2) _IXOSC 60S acidic ribosomal protein P0, putative (EC 4.... [47](http://www.expasy.ch/cgi-bin/blast.pl#A24%23A24) 2e-04

tr [B4QKR0](http://www.uniprot.org/uniprot/B4QKR0) _DROSI GD15019 [GD15019] [Drosophila simulans (Fruit fly)] [47](http://www.expasy.ch/cgi-bin/blast.pl#A25%23A25) 2e-04

tr [B4PI59](http://www.uniprot.org/uniprot/B4PI59) _DROYA RpLP0 [Dyak\RpLP0] [Drosophila yakuba (Fruit fly)] [47](http://www.expasy.ch/cgi-bin/blast.pl#A26%23A26) 2e-04

tr [B4N4U1](http://www.uniprot.org/uniprot/B4N4U1) _DROWI GK20443 [GK20443] [Drosophila willistoni (Fruit ... [47](http://www.expasy.ch/cgi-bin/blast.pl#A27%23A27) 2e-04

tr [B4LE06](http://www.uniprot.org/uniprot/B4LE06) _DROVI GJ13582 [GJ13582] [Drosophila virilis (Fruit fly)] [47](http://www.expasy.ch/cgi-bin/blast.pl#A28%23A28) 2e-04

tr [B4KV97](http://www.uniprot.org/uniprot/B4KV97) _DROMO GI13777 [GI13777] [Drosophila mojavensis (Fruit ... [47](http://www.expasy.ch/cgi-bin/blast.pl#A29%23A29) 2e-04

tr [B4IXN3](http://www.uniprot.org/uniprot/B4IXN3) _DROGR GH14667 [GH14667] [Drosophila grimshawi (Fruit f... [47](http://www.expasy.ch/cgi-bin/blast.pl#A30%23A30) 2e-04

tr [B4IAY7](http://www.uniprot.org/uniprot/B4IAY7) _DROSE GM22429 [GM22429] [Drosophila sechellia (Fruit f... [47](http://www.expasy.ch/cgi-bin/blast.pl#A31%23A31) 2e-04

tr [B3NJ27](http://www.uniprot.org/uniprot/B3NJ27) _DROER GG16244 [GG16244] [Drosophila erecta (Fruit fly)] [47](http://www.expasy.ch/cgi-bin/blast.pl#A32%23A32) 2e-04

tr [B3MAK8](http://www.uniprot.org/uniprot/B3MAK8) _DROAN GF10946 [GF10946] [Drosophila ananassae (Fruit f... [47](http://www.expasy.ch/cgi-bin/blast.pl#A33%23A33) 2e-04

tr [A9Y1V1](http://www.uniprot.org/uniprot/A9Y1V1) _HAELO Ribosomal protein P0 [Haemaphysalis longicornis ... [47](http://www.expasy.ch/cgi-bin/blast.pl#A34%23A34) 2e-04

tr [A7TS21](http://www.uniprot.org/uniprot/A7TS21) _VANPO Putative uncharacterized protein [Kpol_385p9] [V... [47](http://www.expasy.ch/cgi-bin/blast.pl#A35%23A35) 2e-04

sp [P51408](http://www.uniprot.org/uniprot/P51408) RLA2_TRYBB 60S acidic ribosomal protein P2 [Trypanosom... [45](http://www.expasy.ch/cgi-bin/blast.pl#A36%23A36) 0.001

sp [P02399](http://www.uniprot.org/uniprot/P02399) RLA2_ARTSA 60S acidic ribosomal protein P2 (EL12) [Art... [45](http://www.expasy.ch/cgi-bin/blast.pl#A37%23A37) 0.001

sp [P02402](http://www.uniprot.org/uniprot/P02402) RLA1_ARTSA 60S acidic ribosomal protein P1 (eL12'/ eL1... [45](http://www.expasy.ch/cgi-bin/blast.pl#A38%23A38) 0.001

tr [Q586I4](http://www.uniprot.org/uniprot/Q586I4) _9TRYP 60S acidic ribosomal protein P2, putative [Tb927... [45](http://www.expasy.ch/cgi-bin/blast.pl#A39%23A39) 0.001

tr [Q57ZQ1](http://www.uniprot.org/uniprot/Q57ZQ1) _9TRYP 60S acidic ribosomal protein, putative [Tb927.5.... [45](http://www.expasy.ch/cgi-bin/blast.pl#A40%23A40) 0.001

tr [Q38BQ9](http://www.uniprot.org/uniprot/Q38BQ9) _9TRYP 60S acidic ribosomal protein P2, putative [Tb10.... [45](http://www.expasy.ch/cgi-bin/blast.pl#A41%23A41) 0.001

tr [C4WU73](http://www.uniprot.org/uniprot/C4WU73) _ACYPI ACYPI002961 protein [ACYPI002961] [Acyrthosiphon... [45](http://www.expasy.ch/cgi-bin/blast.pl#A42%23A42) 0.001

tr [A1DU40](http://www.uniprot.org/uniprot/A1DU40) _ARTSF 60S acidic ribosomal protein P2 [Artemia sanfran... [45](http://www.expasy.ch/cgi-bin/blast.pl#A43%23A43) 0.001

tr [A1KXI7](http://www.uniprot.org/uniprot/A1KXI7) _BLOTA Blo t Alt a 6 allergen [Blomia tropicalis (Mite)] [44](http://www.expasy.ch/cgi-bin/blast.pl#A44%23A44) 0.001

sp [Q9U3U0](http://www.uniprot.org/uniprot/Q9U3U0) RLA0_CERCA 60S acidic ribosomal protein P0 (CcP0) [RpL... [44](http://www.expasy.ch/cgi-bin/blast.pl#A45%23A45) 0.002

tr [Q4PMB4](http://www.uniprot.org/uniprot/Q4PMB4) _IXOSC 60S acidic ribosomal protein P0 [Ixodes scapular... [44](http://www.expasy.ch/cgi-bin/blast.pl#A46%23A46) 0.002

tr [C1PHC5](http://www.uniprot.org/uniprot/C1PHC5) _BABRO 60S acidic ribosomal protein P0 (Fragment) [Babe... [44](http://www.expasy.ch/cgi-bin/blast.pl#A47%23A47) 0.002

tr [B2ZRT0](http://www.uniprot.org/uniprot/B2ZRT0) _9ACAR Ribosomal protein [LA-2] [Haemaphysalis qinghaie... [44](http://www.expasy.ch/cgi-bin/blast.pl#A48%23A48) 0.002

tr [C4QYM0](http://www.uniprot.org/uniprot/C4QYM0) _PICPG 60S acidic ribosomal protein P2-A [PAS_chr1-4_04... [44](http://www.expasy.ch/cgi-bin/blast.pl#A49%23A49) 0.002

tr [A7TPM0](http://www.uniprot.org/uniprot/A7TPM0) _VANPO Putative uncharacterized protein [Kpol_1040p25] ... [44](http://www.expasy.ch/cgi-bin/blast.pl#A50%23A50) 0.002

tr [A7TF23](http://www.uniprot.org/uniprot/A7TF23) _VANPO Putative uncharacterized protein [Kpol_2000p12] ... [44](http://www.expasy.ch/cgi-bin/blast.pl#A51%23A51) 0.002

tr [A7KMW4](http://www.uniprot.org/uniprot/A7KMW4) _9BASI 60S acidic ribosomal protein P1 [Melampsora medu... [43](http://www.expasy.ch/cgi-bin/blast.pl#A52%23A52) 0.003

sp [Q06382](http://www.uniprot.org/uniprot/Q06382) RLA3_LEIIN 60S acidic ribosomal protein P2-2 [LIP'] [L... [43](http://www.expasy.ch/cgi-bin/blast.pl#A53%23A53) 0.003

sp [Q06383](http://www.uniprot.org/uniprot/Q06383) RLA2_LEIIN 60S acidic ribosomal protein P2-1 [LIP] [Le... [43](http://www.expasy.ch/cgi-bin/blast.pl#A54%23A54) 0.003

sp [O43940](http://www.uniprot.org/uniprot/O43940) RLA2_LEIDO 60S acidic ribosomal protein P2 (Acidic rib... [43](http://www.expasy.ch/cgi-bin/blast.pl#A55%23A55) 0.003

sp [O44010](http://www.uniprot.org/uniprot/O44010) RLA2_LEIBR 60S acidic ribosomal protein P2 (Acidic rib... [43](http://www.expasy.ch/cgi-bin/blast.pl#A56%23A56) 0.003

sp [O61463](http://www.uniprot.org/uniprot/O61463) RLA2_CRYST 60S acidic ribosomal protein P2 [Cryptochit... [43](http://www.expasy.ch/cgi-bin/blast.pl#A57%23A57) 0.003

sp [O01725](http://www.uniprot.org/uniprot/O01725) RLA2_BRAFL 60S acidic ribosomal protein P2 [Branchiost... [43](http://www.expasy.ch/cgi-bin/blast.pl#A58%23A58) 0.003

sp [O46313](http://www.uniprot.org/uniprot/O46313) RLA1_LEIPE 60S acidic ribosomal protein P1 [Leishmania... [43](http://www.expasy.ch/cgi-bin/blast.pl#A59%23A59) 0.003

sp [P05317](http://www.uniprot.org/uniprot/P05317) RLA0_YEAST 60S acidic ribosomal protein P0 (A0) (L10E)... [43](http://www.expasy.ch/cgi-bin/blast.pl#A60%23A60) 0.003

tr [Q9N6F4](http://www.uniprot.org/uniprot/Q9N6F4) _LEIMA 60S acidic ribosomal protein P2, putative [LMJ_0... [43](http://www.expasy.ch/cgi-bin/blast.pl#A61%23A61) 0.003

tr [Q8ISP6](http://www.uniprot.org/uniprot/Q8ISP6) _BRABE Ribosomal protein P1 [Branchiostoma belcheri (Am... [43](http://www.expasy.ch/cgi-bin/blast.pl#A62%23A62) 0.003

tr [Q8I867](http://www.uniprot.org/uniprot/Q8I867) _LEIDO Ribosomal protein P1-like protein [Leishmania do... [43](http://www.expasy.ch/cgi-bin/blast.pl#A63%23A63) 0.003

tr [Q70YZ3](http://www.uniprot.org/uniprot/Q70YZ3) _BOMMO Ribosomal P0 protein [Bombyx mori (Silk moth)] [43](http://www.expasy.ch/cgi-bin/blast.pl#A64%23A64) 0.003

tr [Q66VD2](http://www.uniprot.org/uniprot/Q66VD2) _LEIDO Acidic ribosomal protein [Leishmania donovani] [43](http://www.expasy.ch/cgi-bin/blast.pl#A65%23A65) 0.003

tr [Q5UAU1](http://www.uniprot.org/uniprot/Q5UAU1) _BOMMO Ribosomal protein P0 [RpP0] [Bombyx mori (Silk m... [43](http://www.expasy.ch/cgi-bin/blast.pl#A66%23A66) 0.003

tr [Q4QF62](http://www.uniprot.org/uniprot/Q4QF62) _LEIMA 60S acidic ribosomal protein P2 [LmjF15.1203] [L... [43](http://www.expasy.ch/cgi-bin/blast.pl#A67%23A67) 0.003

tr [Q4Q6R6](http://www.uniprot.org/uniprot/Q4Q6R6) _LEIMA 60S acidic ribosomal protein P2, putative [LmjF3... [43](http://www.expasy.ch/cgi-bin/blast.pl#A68%23A68) 0.003

tr [Q4Q6R5](http://www.uniprot.org/uniprot/Q4Q6R5) _LEIMA 60S acidic ribosomal protein P2, putative [LmjF3... [43](http://www.expasy.ch/cgi-bin/blast.pl#A69%23A69) 0.003

tr [Q4KTI8](http://www.uniprot.org/uniprot/Q4KTI8) _SUBDO P1 [Suberites domuncula (Sponge)] [43](http://www.expasy.ch/cgi-bin/blast.pl#A70%23A70) 0.003

tr [Q4KTI7](http://www.uniprot.org/uniprot/Q4KTI7) _SUBDO P2 [Suberites domuncula (Sponge)] [43](http://www.expasy.ch/cgi-bin/blast.pl#A71%23A71) 0.003

tr [Q4KTH7](http://www.uniprot.org/uniprot/Q4KTH7) _SUBDO L10e/P0 [Suberites domuncula (Sponge)] [43](http://www.expasy.ch/cgi-bin/blast.pl#A72%23A72) 0.003

tr [Q1HCM5](http://www.uniprot.org/uniprot/Q1HCM5) _PECGU Ribosomal protein P1 [Pectinaria gouldii (Trumpe... [43](http://www.expasy.ch/cgi-bin/blast.pl#A73%23A73) 0.003

tr [C3ZD79](http://www.uniprot.org/uniprot/C3ZD79) _BRAFL Putative uncharacterized protein [BRAFLDRAFT_206... [43](http://www.expasy.ch/cgi-bin/blast.pl#A74%23A74) 0.003

tr [C3Z9A6](http://www.uniprot.org/uniprot/C3Z9A6) _BRAFL Putative uncharacterized protein [BRAFLDRAFT_124... [43](http://www.expasy.ch/cgi-bin/blast.pl#A75%23A75) 0.003

tr [C3Z0M5](http://www.uniprot.org/uniprot/C3Z0M5) _BRAFL Putative uncharacterized protein [BRAFLDRAFT_978... [43](http://www.expasy.ch/cgi-bin/blast.pl#A76%23A76) 0.003

tr [C1BV18](http://www.uniprot.org/uniprot/C1BV18) _9MAXI 60S acidic ribosomal protein P2 [RLA2] [Lepeopht... [43](http://www.expasy.ch/cgi-bin/blast.pl#A77%23A77) 0.003

tr [C1BTD7](http://www.uniprot.org/uniprot/C1BTD7) _9MAXI 60S acidic ribosomal protein P0 [RLA0] [Lepeopht... [43](http://www.expasy.ch/cgi-bin/blast.pl#A78%23A78) 0.003

tr [B6ZCB2](http://www.uniprot.org/uniprot/B6ZCB2) _MYTGA Ribosomal protein [p0] [Mytilus galloprovinciali... [43](http://www.expasy.ch/cgi-bin/blast.pl#A79%23A79) 0.003

tr [B4XPB7](http://www.uniprot.org/uniprot/B4XPB7) _9CNID 60S acidic ribosomal phosphoprotein P0 [36B4] [S... [43](http://www.expasy.ch/cgi-bin/blast.pl#A80%23A80) 0.003

tr [B3S429](http://www.uniprot.org/uniprot/B3S429) _TRIAD Putative uncharacterized protein [TRIADDRAFT_633... [43](http://www.expasy.ch/cgi-bin/blast.pl#A81%23A81) 0.003

tr [B3S1Z8](http://www.uniprot.org/uniprot/B3S1Z8) _TRIAD Putative uncharacterized protein [TRIADDRAFT_631... [43](http://www.expasy.ch/cgi-bin/blast.pl#A82%23A82) 0.003

tr [B3S1V3](http://www.uniprot.org/uniprot/B3S1V3) _TRIAD Putative uncharacterized protein [TRIADDRAFT_379... [43](http://www.expasy.ch/cgi-bin/blast.pl#A83%23A83) 0.003

tr [B3GQX2](http://www.uniprot.org/uniprot/B3GQX2) _ASTPE 60S acidic ribosomal protein P2 [Asterina pectin... [43](http://www.expasy.ch/cgi-bin/blast.pl#A84%23A84) 0.003

tr [B0Z9P2](http://www.uniprot.org/uniprot/B0Z9P2) _9BILA Ribosomal protein rplp2 [Lineus viridis] [43](http://www.expasy.ch/cgi-bin/blast.pl#A85%23A85) 0.003

tr [B0Z9P1](http://www.uniprot.org/uniprot/B0Z9P1) _9BILA Ribosomal protein rplp1 [Lineus viridis] [43](http://www.expasy.ch/cgi-bin/blast.pl#A86%23A86) 0.003

tr [B0Z9P0](http://www.uniprot.org/uniprot/B0Z9P0) _9BILA Ribosomal protein rplp0 [Lineus viridis] [43](http://www.expasy.ch/cgi-bin/blast.pl#A87%23A87) 0.003

tr [A7SQ36](http://www.uniprot.org/uniprot/A7SQ36) _NEMVE Predicted protein [v1g237425] [Nematostella vect... [43](http://www.expasy.ch/cgi-bin/blast.pl#A88%23A88) 0.003

tr [A7S837](http://www.uniprot.org/uniprot/A7S837) _NEMVE Predicted protein [v1g186829] [Nematostella vect... [43](http://www.expasy.ch/cgi-bin/blast.pl#A89%23A89) 0.003

tr [A7RLY2](http://www.uniprot.org/uniprot/A7RLY2) _NEMVE Predicted protein [v1g160310] [Nematostella vect... [43](http://www.expasy.ch/cgi-bin/blast.pl#A90%23A90) 0.003

tr [A4I600](http://www.uniprot.org/uniprot/A4I600) _LEIIN 60S acidic ribosomal protein P2, putative [LinJ3... [43](http://www.expasy.ch/cgi-bin/blast.pl#A91%23A91) 0.003

tr [A4I5Z9](http://www.uniprot.org/uniprot/A4I5Z9) _LEIIN 60S acidic ribosomal protein P2, putative [LinJ3... [43](http://www.expasy.ch/cgi-bin/blast.pl#A92%23A92) 0.003

tr [A4HRV4](http://www.uniprot.org/uniprot/A4HRV4) _LEIIN 60S acidic ribosomal protein P2, putative [LinJ0... [43](http://www.expasy.ch/cgi-bin/blast.pl#A93%23A93) 0.003

tr [A4HIV9](http://www.uniprot.org/uniprot/A4HIV9) _LEIBR 60S acidic ribosomal protein P2, putative [LbrM3... [43](http://www.expasy.ch/cgi-bin/blast.pl#A94%23A94) 0.003

tr [A4H884](http://www.uniprot.org/uniprot/A4H884) _LEIBR 60S acidic ribosomal protein P2 [LbrM15_V2.1150]... [43](http://www.expasy.ch/cgi-bin/blast.pl#A95%23A95) 0.003

tr [A4H3L1](http://www.uniprot.org/uniprot/A4H3L1) _LEIBR 60S acidic ribosomal protein P2, putative [LbrM0... [43](http://www.expasy.ch/cgi-bin/blast.pl#A96%23A96) 0.003

tr [A4H3L0](http://www.uniprot.org/uniprot/A4H3L0) _LEIBR 60S acidic ribosomal protein P2, putative [LbrM0... [43](http://www.expasy.ch/cgi-bin/blast.pl#A97%23A97) 0.003

tr [A2I3Y7](http://www.uniprot.org/uniprot/A2I3Y7) _MACHI 60S acidic ribosomal protein P1-like protein [Ma... [43](http://www.expasy.ch/cgi-bin/blast.pl#A98%23A98) 0.003

tr [Q75CU7](http://www.uniprot.org/uniprot/Q75CU7) _ASHGO ACL178Cp [ACL178C] [Ashbya gossypii (Yeast) (Ere... [43](http://www.expasy.ch/cgi-bin/blast.pl#A99%23A99) 0.003

tr [Q6CW89](http://www.uniprot.org/uniprot/Q6CW89) _KLULA KLLA0B05918p [KLLA0B05918g] [Kluyveromyces lacti... [43](http://www.expasy.ch/cgi-bin/blast.pl#A100%23A100) 0.003

Bottom of Form

**Graphical overview of the alignments**


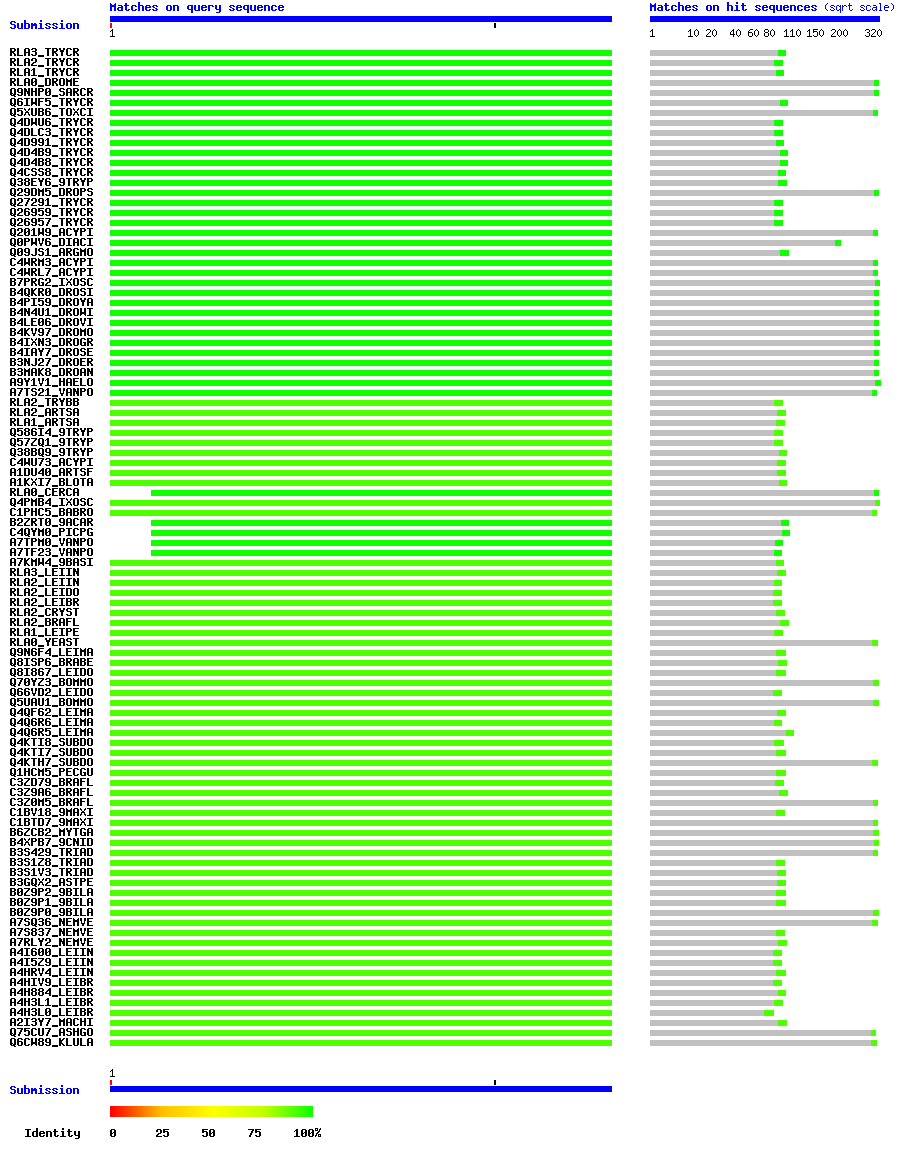


**Alignments**

| | | sp | [P26795](http://www.uniprot.org/uniprot/P26795) RLA3_TRYCR | **60S acidic ribosomal protein P2-B (P2B) [Trypanosoma cruzi]** | 112 AA | | --- | --- | --- | --- | | Score = 47.3 bits (104), Expect = 2e-04  Identities = 13/13 (100%), Positives = 13/13 (100%)  Query: 1 EEEDDDMGFGLFD 13  EEEDDDMGFGLFD  Sbjct: 100 EEEDDDMGFGLFD 112 | | | | | | --- | --- | --- | --- | --- | --- | --- | --- | --- | |
| --- | --- | --- | --- | --- | --- | --- | --- | --- | --- |

| | | sp | [P23632](http://www.uniprot.org/uniprot/P23632) RLA2_TRYCR | **60S acidic ribosomal protein P2-A (P) (P-JL5) (L12E) [Trypanosoma cruzi]** | 107 AA | | --- | --- | --- | --- | | Score = 47.3 bits (104), Expect = 2e-04  Identities = 13/13 (100%), Positives = 13/13 (100%)  Query: 1 EEEDDDMGFGLFD 13  EEEDDDMGFGLFD  Sbjct: 95 EEEDDDMGFGLFD 107 | | | | | | --- | --- | --- | --- | --- | --- | --- | --- | --- | |
| --- | --- | --- | --- | --- | --- | --- | --- | --- | --- |

| | | sp | [P26643](http://www.uniprot.org/uniprot/P26643) RLA1_TRYCR | **60S acidic ribosomal protein P1 [Trypanosoma cruzi]** | 109 AA | | --- | --- | --- | --- | | Score = 47.3 bits (104), Expect = 2e-04  Identities = 13/13 (100%), Positives = 13/13 (100%)  Query: 1 EEEDDDMGFGLFD 13  EEEDDDMGFGLFD  Sbjct: 97 EEEDDDMGFGLFD 109 | | | | | | --- | --- | --- | --- | --- | --- | --- | --- | --- | |
| --- | --- | --- | --- | --- | --- | --- | --- | --- | --- |

| | | sp | [P19889](http://www.uniprot.org/uniprot/P19889) RLA0_DROME | **60S acidic ribosomal protein P0 (DNA-(apurinic or apyrimidinic site) lyase) (EC 4.2.99.18) (Apurinic-apyrimidinic endonuclease) [RpLP0] [Drosophila melanogaster (Fruit fly)]** | 317 AA | | --- | --- | --- | --- | | Score = 47.3 bits (104), Expect = 2e-04  Identities = 13/13 (100%), Positives = 13/13 (100%)  Query: 1 EEEDDDMGFGLFD 13  EEEDDDMGFGLFD  Sbjct: 305 EEEDDDMGFGLFD 317 | | | | | | --- | --- | --- | --- | --- | --- | --- | --- | --- | |
| --- | --- | --- | --- | --- | --- | --- | --- | --- | --- |

| | | tr | [Q9NHP0](http://www.uniprot.org/uniprot/Q9NHP0) Q9NHP0_SARCR | **60S acidic ribosomal protein P0 [Sarcophaga crassipalpis]** | 316 AA | | --- | --- | --- | --- | | Score = 47.3 bits (104), Expect = 2e-04  Identities = 13/13 (100%), Positives = 13/13 (100%)  Query: 1 EEEDDDMGFGLFD 13  EEEDDDMGFGLFD  Sbjct: 304 EEEDDDMGFGLFD 316 | | | | | | --- | --- | --- | --- | --- | --- | --- | --- | --- | |
| --- | --- | --- | --- | --- | --- | --- | --- | --- | --- |

| | | tr | [Q6IWF5](http://www.uniprot.org/uniprot/Q6IWF5) Q6IWF5_TRYCR | **Ribosomal P protein type 1 (60S acidic ribosomal protein P2, putative) [Tc00.1047053503505.10] [Trypanosoma cruzi]** | 115 AA | | --- | --- | --- | --- | | Score = 47.3 bits (104), Expect = 2e-04  Identities = 13/13 (100%), Positives = 13/13 (100%)  Query: 1 EEEDDDMGFGLFD 13  EEEDDDMGFGLFD  Sbjct: 103 EEEDDDMGFGLFD 115 | | | | | | --- | --- | --- | --- | --- | --- | --- | --- | --- | |
| --- | --- | --- | --- | --- | --- | --- | --- | --- | --- |

| | | tr | [Q5XUB6](http://www.uniprot.org/uniprot/Q5XUB6) Q5XUB6_TOXCI | **Putative acidic p0 ribosomal protein [Toxoptera citricida (Brown citrus aphid)]** | 314 AA | | --- | --- | --- | --- | | Score = 47.3 bits (104), Expect = 2e-04  Identities = 13/13 (100%), Positives = 13/13 (100%)  Query: 1 EEEDDDMGFGLFD 13  EEEDDDMGFGLFD  Sbjct: 302 EEEDDDMGFGLFD 314 | | | | | | --- | --- | --- | --- | --- | --- | --- | --- | --- | |
| --- | --- | --- | --- | --- | --- | --- | --- | --- | --- |

| | | tr | [Q4DWU6](http://www.uniprot.org/uniprot/Q4DWU6) Q4DWU6_TRYCR | **60S acidic ribosomal protein P2, putative [Tc00.1047053510643.140] [Trypanosoma cruzi]** | 107 AA | | --- | --- | --- | --- | | Score = 47.3 bits (104), Expect = 2e-04  Identities = 13/13 (100%), Positives = 13/13 (100%)  Query: 1 EEEDDDMGFGLFD 13  EEEDDDMGFGLFD  Sbjct: 95 EEEDDDMGFGLFD 107 | | | | | | --- | --- | --- | --- | --- | --- | --- | --- | --- | |
| --- | --- | --- | --- | --- | --- | --- | --- | --- | --- |

| | | tr | [Q4DLC3](http://www.uniprot.org/uniprot/Q4DLC3) Q4DLC3_TRYCR | **60S acidic ribosomal protein P2, putative [Tc00.1047053506595.100] [Trypanosoma cruzi]** | 107 AA | | --- | --- | --- | --- | | Score = 47.3 bits (104), Expect = 2e-04  Identities = 13/13 (100%), Positives = 13/13 (100%)  Query: 1 EEEDDDMGFGLFD 13  EEEDDDMGFGLFD  Sbjct: 95 EEEDDDMGFGLFD 107 | | | | | | --- | --- | --- | --- | --- | --- | --- | --- | --- | |
| --- | --- | --- | --- | --- | --- | --- | --- | --- | --- |

| | | tr | [Q4D991](http://www.uniprot.org/uniprot/Q4D991) Q4D991_TRYCR | **60S acidic ribosomal protein, putative [Tc00.1047053510823.70] [Trypanosoma cruzi]** | 109 AA | | --- | --- | --- | --- | | Score = 47.3 bits (104), Expect = 2e-04  Identities = 13/13 (100%), Positives = 13/13 (100%)  Query: 1 EEEDDDMGFGLFD 13  EEEDDDMGFGLFD  Sbjct: 97 EEEDDDMGFGLFD 109 | | | | | | --- | --- | --- | --- | --- | --- | --- | --- | --- | |
| --- | --- | --- | --- | --- | --- | --- | --- | --- | --- |

| | | tr | [Q4D4B9](http://www.uniprot.org/uniprot/Q4D4B9) Q4D4B9_TRYCR | **60S acidic ribosomal protein P2, putative [Tc00.1047053510309.40] [Trypanosoma cruzi]** | 115 AA | | --- | --- | --- | --- | | Score = 47.3 bits (104), Expect = 2e-04  Identities = 13/13 (100%), Positives = 13/13 (100%)  Query: 1 EEEDDDMGFGLFD 13  EEEDDDMGFGLFD  Sbjct: 103 EEEDDDMGFGLFD 115 | | | | | | --- | --- | --- | --- | --- | --- | --- | --- | --- | |
| --- | --- | --- | --- | --- | --- | --- | --- | --- | --- |

| | | tr | [Q4D4B8](http://www.uniprot.org/uniprot/Q4D4B8) Q4D4B8_TRYCR | **60S acidic ribosomal protein P2, putative [Tc00.1047053510309.50] [Trypanosoma cruzi]** | 115 AA | | --- | --- | --- | --- | | Score = 47.3 bits (104), Expect = 2e-04  Identities = 13/13 (100%), Positives = 13/13 (100%)  Query: 1 EEEDDDMGFGLFD 13  EEEDDDMGFGLFD  Sbjct: 103 EEEDDDMGFGLFD 115 | | | | | | --- | --- | --- | --- | --- | --- | --- | --- | --- | |
| --- | --- | --- | --- | --- | --- | --- | --- | --- | --- |

| | | tr | [Q4CSS8](http://www.uniprot.org/uniprot/Q4CSS8) Q4CSS8_TRYCR | **60S acidic ribosomal protein P2, putative [Tc00.1047053505977.26] [Trypanosoma cruzi]** | 112 AA | | --- | --- | --- | --- | | Score = 47.3 bits (104), Expect = 2e-04  Identities = 13/13 (100%), Positives = 13/13 (100%)  Query: 1 EEEDDDMGFGLFD 13  EEEDDDMGFGLFD  Sbjct: 100 EEEDDDMGFGLFD 112 | | | | | | --- | --- | --- | --- | --- | --- | --- | --- | --- | |
| --- | --- | --- | --- | --- | --- | --- | --- | --- | --- |

| | | tr | [Q38EY6](http://www.uniprot.org/uniprot/Q38EY6) Q38EY6_9TRYP | **60S acidic ribosomal protein, putative [Tb09.160.4200] [Trypanosoma brucei]** | 113 AA | | --- | --- | --- | --- | | Score = 47.3 bits (104), Expect = 2e-04  Identities = 13/13 (100%), Positives = 13/13 (100%)  Query: 1 EEEDDDMGFGLFD 13  EEEDDDMGFGLFD  Sbjct: 101 EEEDDDMGFGLFD 113 | | | | | | --- | --- | --- | --- | --- | --- | --- | --- | --- | |
| --- | --- | --- | --- | --- | --- | --- | --- | --- | --- |

| | | tr | [Q29DM5](http://www.uniprot.org/uniprot/Q29DM5) Q29DM5_DROPS | **GA20389 [GA20389] [Drosophila pseudoobscura pseudoobscura (Fruit fly)]** | 317 AA | | --- | --- | --- | --- | | Score = 47.3 bits (104), Expect = 2e-04  Identities = 13/13 (100%), Positives = 13/13 (100%)  Query: 1 EEEDDDMGFGLFD 13  EEEDDDMGFGLFD  Sbjct: 305 EEEDDDMGFGLFD 317 | | | | | | --- | --- | --- | --- | --- | --- | --- | --- | --- | |
| --- | --- | --- | --- | --- | --- | --- | --- | --- | --- |

| | | tr | [Q27291](http://www.uniprot.org/uniprot/Q27291) Q27291_TRYCR | **TcP2beta protein [TcP2beta] [Trypanosoma cruzi]** | 107 AA | | --- | --- | --- | --- | | Score = 47.3 bits (104), Expect = 2e-04  Identities = 13/13 (100%), Positives = 13/13 (100%)  Query: 1 EEEDDDMGFGLFD 13  EEEDDDMGFGLFD  Sbjct: 95 EEEDDDMGFGLFD 107 | | | | | | --- | --- | --- | --- | --- | --- | --- | --- | --- | |
| --- | --- | --- | --- | --- | --- | --- | --- | --- | --- |

| | | tr | [Q26959](http://www.uniprot.org/uniprot/Q26959) Q26959_TRYCR | **TcP2beta protein [TcP2beta] [Trypanosoma cruzi]** | 107 AA | | --- | --- | --- | --- | | Score = 47.3 bits (104), Expect = 2e-04  Identities = 13/13 (100%), Positives = 13/13 (100%)  Query: 1 EEEDDDMGFGLFD 13  EEEDDDMGFGLFD  Sbjct: 95 EEEDDDMGFGLFD 107 | | | | | | --- | --- | --- | --- | --- | --- | --- | --- | --- | |
| --- | --- | --- | --- | --- | --- | --- | --- | --- | --- |

| | | tr | [Q26957](http://www.uniprot.org/uniprot/Q26957) Q26957_TRYCR | **60S acidic ribosomal protein P2 beta (H6.4), putative (TcP2beta protein) [TcP2beta] [Trypanosoma cruzi]** | 107 AA | | --- | --- | --- | --- | | Score = 47.3 bits (104), Expect = 2e-04  Identities = 13/13 (100%), Positives = 13/13 (100%)  Query: 1 EEEDDDMGFGLFD 13  EEEDDDMGFGLFD  Sbjct: 95 EEEDDDMGFGLFD 107 | | | | | | --- | --- | --- | --- | --- | --- | --- | --- | --- | |
| --- | --- | --- | --- | --- | --- | --- | --- | --- | --- |

| | | tr | [Q201W9](http://www.uniprot.org/uniprot/Q201W9) Q201W9_ACYPI | **ACYPI000079 protein (Putative acidic p0 ribosomal protein) [ACYPI000079] [Acyrthosiphon pisum (Pea aphid)]** | 314 AA | | --- | --- | --- | --- | | Score = 47.3 bits (104), Expect = 2e-04  Identities = 13/13 (100%), Positives = 13/13 (100%)  Query: 1 EEEDDDMGFGLFD 13  EEEDDDMGFGLFD  Sbjct: 302 EEEDDDMGFGLFD 314 | | | | | | --- | --- | --- | --- | --- | --- | --- | --- | --- | |
| --- | --- | --- | --- | --- | --- | --- | --- | --- | --- |

| | | tr | [Q0PWV6](http://www.uniprot.org/uniprot/Q0PWV6) Q0PWV6_DIACI | **Putative acidic p0 ribosomal protein (Fragment) [Diaphorina citri (Asian citrus psyllid)]** | 220 AA | | --- | --- | --- | --- | | Score = 47.3 bits (104), Expect = 2e-04  Identities = 13/13 (100%), Positives = 13/13 (100%)  Query: 1 EEEDDDMGFGLFD 13  EEEDDDMGFGLFD  Sbjct: 208 EEEDDDMGFGLFD 220 | | | | | | --- | --- | --- | --- | --- | --- | --- | --- | --- | |
| --- | --- | --- | --- | --- | --- | --- | --- | --- | --- |

| | | tr | [Q09JS1](http://www.uniprot.org/uniprot/Q09JS1) Q09JS1_ARGMO | **Ribosomal protein LP1 [Argas monolakensis (Mono lake bird tick)]** | 116 AA | | --- | --- | --- | --- | | Score = 47.3 bits (104), Expect = 2e-04  Identities = 13/13 (100%), Positives = 13/13 (100%)  Query: 1 EEEDDDMGFGLFD 13  EEEDDDMGFGLFD  Sbjct: 104 EEEDDDMGFGLFD 116 | | | | | | --- | --- | --- | --- | --- | --- | --- | --- | --- | |
| --- | --- | --- | --- | --- | --- | --- | --- | --- | --- |

| | | tr | [C4WRM3](http://www.uniprot.org/uniprot/C4WRM3) C4WRM3_ACYPI | **ACYPI000079 protein [ACYPI000079] [Acyrthosiphon pisum (Pea aphid)]** | 314 AA | | --- | --- | --- | --- | | Score = 47.3 bits (104), Expect = 2e-04  Identities = 13/13 (100%), Positives = 13/13 (100%)  Query: 1 EEEDDDMGFGLFD 13  EEEDDDMGFGLFD  Sbjct: 302 EEEDDDMGFGLFD 314 | | | | | | --- | --- | --- | --- | --- | --- | --- | --- | --- | |
| --- | --- | --- | --- | --- | --- | --- | --- | --- | --- |

| | | tr | [C4WRL7](http://www.uniprot.org/uniprot/C4WRL7) C4WRL7_ACYPI | **ACYPI000079 protein [ACYPI000079] [Acyrthosiphon pisum (Pea aphid)]** | 314 AA | | --- | --- | --- | --- | | Score = 47.3 bits (104), Expect = 2e-04  Identities = 13/13 (100%), Positives = 13/13 (100%)  Query: 1 EEEDDDMGFGLFD 13  EEEDDDMGFGLFD  Sbjct: 302 EEEDDDMGFGLFD 314 | | | | | | --- | --- | --- | --- | --- | --- | --- | --- | --- | |
| --- | --- | --- | --- | --- | --- | --- | --- | --- | --- |

| | | tr | [B7PRG2](http://www.uniprot.org/uniprot/B7PRG2) B7PRG2_IXOSC | **60S acidic ribosomal protein P0, putative (EC 4.2.99.18) [IscW_ISCW007303] [Ixodes scapularis (Black-legged tick) (Deer tick)]** | 319 AA | | --- | --- | --- | --- | | Score = 47.3 bits (104), Expect = 2e-04  Identities = 13/13 (100%), Positives = 13/13 (100%)  Query: 1 EEEDDDMGFGLFD 13  EEEDDDMGFGLFD  Sbjct: 307 EEEDDDMGFGLFD 319 | | | | | | --- | --- | --- | --- | --- | --- | --- | --- | --- | |
| --- | --- | --- | --- | --- | --- | --- | --- | --- | --- |

| | | tr | [B4QKR0](http://www.uniprot.org/uniprot/B4QKR0) B4QKR0_DROSI | **GD15019 [GD15019] [Drosophila simulans (Fruit fly)]** | 317 AA | | --- | --- | --- | --- | | Score = 47.3 bits (104), Expect = 2e-04  Identities = 13/13 (100%), Positives = 13/13 (100%)  Query: 1 EEEDDDMGFGLFD 13  EEEDDDMGFGLFD  Sbjct: 305 EEEDDDMGFGLFD 317 | | | | | | --- | --- | --- | --- | --- | --- | --- | --- | --- | |
| --- | --- | --- | --- | --- | --- | --- | --- | --- | --- |

| | | tr | [B4PI59](http://www.uniprot.org/uniprot/B4PI59) B4PI59_DROYA | **RpLP0 [Dyak\RpLP0] [Drosophila yakuba (Fruit fly)]** | 317 AA | | --- | --- | --- | --- | | Score = 47.3 bits (104), Expect = 2e-04  Identities = 13/13 (100%), Positives = 13/13 (100%)  Query: 1 EEEDDDMGFGLFD 13  EEEDDDMGFGLFD  Sbjct: 305 EEEDDDMGFGLFD 317 | | | | | | --- | --- | --- | --- | --- | --- | --- | --- | --- | |
| --- | --- | --- | --- | --- | --- | --- | --- | --- | --- |

| | | tr | [B4N4U1](http://www.uniprot.org/uniprot/B4N4U1) B4N4U1_DROWI | **GK20443 [GK20443] [Drosophila willistoni (Fruit fly)]** | 317 AA | | --- | --- | --- | --- | | Score = 47.3 bits (104), Expect = 2e-04  Identities = 13/13 (100%), Positives = 13/13 (100%)  Query: 1 EEEDDDMGFGLFD 13  EEEDDDMGFGLFD  Sbjct: 305 EEEDDDMGFGLFD 317 | | | | | | --- | --- | --- | --- | --- | --- | --- | --- | --- | |
| --- | --- | --- | --- | --- | --- | --- | --- | --- | --- |

| | | tr | [B4LE06](http://www.uniprot.org/uniprot/B4LE06) B4LE06_DROVI | **GJ13582 [GJ13582] [Drosophila virilis (Fruit fly)]** | 317 AA | | --- | --- | --- | --- | | Score = 47.3 bits (104), Expect = 2e-04  Identities = 13/13 (100%), Positives = 13/13 (100%)  Query: 1 EEEDDDMGFGLFD 13  EEEDDDMGFGLFD  Sbjct: 305 EEEDDDMGFGLFD 317 | | | | | | --- | --- | --- | --- | --- | --- | --- | --- | --- | |
| --- | --- | --- | --- | --- | --- | --- | --- | --- | --- |

| | | tr | [B4KV97](http://www.uniprot.org/uniprot/B4KV97) B4KV97_DROMO | **GI13777 [GI13777] [Drosophila mojavensis (Fruit fly)]** | 317 AA | | --- | --- | --- | --- | | Score = 47.3 bits (104), Expect = 2e-04  Identities = 13/13 (100%), Positives = 13/13 (100%)  Query: 1 EEEDDDMGFGLFD 13  EEEDDDMGFGLFD  Sbjct: 305 EEEDDDMGFGLFD 317 | | | | | | --- | --- | --- | --- | --- | --- | --- | --- | --- | |
| --- | --- | --- | --- | --- | --- | --- | --- | --- | --- |

| | | tr | [B4IXN3](http://www.uniprot.org/uniprot/B4IXN3) B4IXN3_DROGR | **GH14667 [GH14667] [Drosophila grimshawi (Fruit fly) (Idiomyia grimshawi)]** | 318 AA | | --- | --- | --- | --- | | Score = 47.3 bits (104), Expect = 2e-04  Identities = 13/13 (100%), Positives = 13/13 (100%)  Query: 1 EEEDDDMGFGLFD 13  EEEDDDMGFGLFD  Sbjct: 306 EEEDDDMGFGLFD 318 | | | | | | --- | --- | --- | --- | --- | --- | --- | --- | --- | |
| --- | --- | --- | --- | --- | --- | --- | --- | --- | --- |

| | | tr | [B4IAY7](http://www.uniprot.org/uniprot/B4IAY7) B4IAY7_DROSE | **GM22429 [GM22429] [Drosophila sechellia (Fruit fly)]** | 317 AA | | --- | --- | --- | --- | | Score = 47.3 bits (104), Expect = 2e-04  Identities = 13/13 (100%), Positives = 13/13 (100%)  Query: 1 EEEDDDMGFGLFD 13  EEEDDDMGFGLFD  Sbjct: 305 EEEDDDMGFGLFD 317 | | | | | | --- | --- | --- | --- | --- | --- | --- | --- | --- | |
| --- | --- | --- | --- | --- | --- | --- | --- | --- | --- |

| | | tr | [B3NJ27](http://www.uniprot.org/uniprot/B3NJ27) B3NJ27_DROER | **GG16244 [GG16244] [Drosophila erecta (Fruit fly)]** | 317 AA | | --- | --- | --- | --- | | Score = 47.3 bits (104), Expect = 2e-04  Identities = 13/13 (100%), Positives = 13/13 (100%)  Query: 1 EEEDDDMGFGLFD 13  EEEDDDMGFGLFD  Sbjct: 305 EEEDDDMGFGLFD 317 | | | | | | --- | --- | --- | --- | --- | --- | --- | --- | --- | |
| --- | --- | --- | --- | --- | --- | --- | --- | --- | --- |

| | | tr | [B3MAK8](http://www.uniprot.org/uniprot/B3MAK8) B3MAK8_DROAN | **GF10946 [GF10946] [Drosophila ananassae (Fruit fly)]** | 317 AA | | --- | --- | --- | --- | | Score = 47.3 bits (104), Expect = 2e-04  Identities = 13/13 (100%), Positives = 13/13 (100%)  Query: 1 EEEDDDMGFGLFD 13  EEEDDDMGFGLFD  Sbjct: 305 EEEDDDMGFGLFD 317 | | | | | | --- | --- | --- | --- | --- | --- | --- | --- | --- | |
| --- | --- | --- | --- | --- | --- | --- | --- | --- | --- |

| | | tr | [A9Y1V1](http://www.uniprot.org/uniprot/A9Y1V1) A9Y1V1_HAELO | **Ribosomal protein P0 [Haemaphysalis longicornis (Bush tick)]** | 320 AA | | --- | --- | --- | --- | | Score = 47.3 bits (104), Expect = 2e-04  Identities = 13/13 (100%), Positives = 13/13 (100%)  Query: 1 EEEDDDMGFGLFD 13  EEEDDDMGFGLFD  Sbjct: 308 EEEDDDMGFGLFD 320 | | | | | | --- | --- | --- | --- | --- | --- | --- | --- | --- | |
| --- | --- | --- | --- | --- | --- | --- | --- | --- | --- |

| | | tr | [A7TS21](http://www.uniprot.org/uniprot/A7TS21) A7TS21_VANPO | **Putative uncharacterized protein [Kpol_385p9] [Vanderwaltozyma polyspora (strain ATCC 22028 / DSM 70294) (Kluyveromyces polysporus)]** | 311 AA | | --- | --- | --- | --- | | Score = 47.3 bits (104), Expect = 2e-04  Identities = 13/13 (100%), Positives = 13/13 (100%)  Query: 1 EEEDDDMGFGLFD 13  EEEDDDMGFGLFD  Sbjct: 299 EEEDDDMGFGLFD 311 | | | | | | --- | --- | --- | --- | --- | --- | --- | --- | --- | |
| --- | --- | --- | --- | --- | --- | --- | --- | --- | --- |

| | | sp | [P51408](http://www.uniprot.org/uniprot/P51408) RLA2_TRYBB | **60S acidic ribosomal protein P2 [Trypanosoma brucei brucei]** | 107 AA | | --- | --- | --- | --- | | Score = 44.8 bits (98), Expect = 0.001  Identities = 12/13 (92%), Positives = 13/13 (100%)  Query: 1 EEEDDDMGFGLFD 13  EE+DDDMGFGLFD  Sbjct: 95 EEDDDDMGFGLFD 107 | | | | | | --- | --- | --- | --- | --- | --- | --- | --- | --- | |
| --- | --- | --- | --- | --- | --- | --- | --- | --- | --- |

| | | sp | [P02399](http://www.uniprot.org/uniprot/P02399) RLA2_ARTSA | **60S acidic ribosomal protein P2 (EL12) [Artemia salina (Brine shrimp)]** | 111 AA | | --- | --- | --- | --- | | Score = 44.8 bits (98), Expect = 0.001  Identities = 12/13 (92%), Positives = 13/13 (100%)  Query: 1 EEEDDDMGFGLFD 13  EEED+DMGFGLFD  Sbjct: 99 EEEDEDMGFGLFD 111 | | | | | | --- | --- | --- | --- | --- | --- | --- | --- | --- | |
| --- | --- | --- | --- | --- | --- | --- | --- | --- | --- |

| | | sp | [P02402](http://www.uniprot.org/uniprot/P02402) RLA1_ARTSA | **60S acidic ribosomal protein P1 (eL12'/ eL12'-P) [Artemia salina (Brine shrimp)]** | 110 AA | | --- | --- | --- | --- | | Score = 44.8 bits (98), Expect = 0.001  Identities = 12/13 (92%), Positives = 13/13 (100%)  Query: 1 EEEDDDMGFGLFD 13  EEED+DMGFGLFD  Sbjct: 98 EEEDEDMGFGLFD 110 | | | | | | --- | --- | --- | --- | --- | --- | --- | --- | --- | |
| --- | --- | --- | --- | --- | --- | --- | --- | --- | --- |

| | | tr | [Q586I4](http://www.uniprot.org/uniprot/Q586I4) Q586I4_9TRYP | **60S acidic ribosomal protein P2, putative [Tb927.6.5120] [Trypanosoma brucei]** | 107 AA | | --- | --- | --- | --- | | Score = 44.8 bits (98), Expect = 0.001  Identities = 12/13 (92%), Positives = 13/13 (100%)  Query: 1 EEEDDDMGFGLFD 13  EE+DDDMGFGLFD  Sbjct: 95 EEDDDDMGFGLFD 107 | | | | | | --- | --- | --- | --- | --- | --- | --- | --- | --- | |
| --- | --- | --- | --- | --- | --- | --- | --- | --- | --- |

| | | tr | [Q57ZQ1](http://www.uniprot.org/uniprot/Q57ZQ1) Q57ZQ1_9TRYP | **60S acidic ribosomal protein, putative [Tb927.5.1820] [Trypanosoma brucei]** | 107 AA | | --- | --- | --- | --- | | Score = 44.8 bits (98), Expect = 0.001  Identities = 12/13 (92%), Positives = 13/13 (100%)  Query: 1 EEEDDDMGFGLFD 13  EE+DDDMGFGLFD  Sbjct: 95 EEDDDDMGFGLFD 107 | | | | | | --- | --- | --- | --- | --- | --- | --- | --- | --- | |
| --- | --- | --- | --- | --- | --- | --- | --- | --- | --- |

| | | tr | [Q38BQ9](http://www.uniprot.org/uniprot/Q38BQ9) Q38BQ9_9TRYP | **60S acidic ribosomal protein P2, putative [Tb10.70.4060] [Trypanosoma brucei]** | 114 AA | | --- | --- | --- | --- | | Score = 44.8 bits (98), Expect = 0.001  Identities = 12/13 (92%), Positives = 13/13 (100%)  Query: 1 EEEDDDMGFGLFD 13  EE+DDDMGFGLFD  Sbjct: 102 EEDDDDMGFGLFD 114 | | | | | | --- | --- | --- | --- | --- | --- | --- | --- | --- | |
| --- | --- | --- | --- | --- | --- | --- | --- | --- | --- |

| | | tr | [C4WU73](http://www.uniprot.org/uniprot/C4WU73) C4WU73_ACYPI | **ACYPI002961 protein [ACYPI002961] [Acyrthosiphon pisum (Pea aphid)]** | 111 AA | | --- | --- | --- | --- | | Score = 44.8 bits (98), Expect = 0.001  Identities = 12/13 (92%), Positives = 13/13 (100%)  Query: 1 EEEDDDMGFGLFD 13  EEEDDDMGFGLF+  Sbjct: 99 EEEDDDMGFGLFE 111 | | | | | | --- | --- | --- | --- | --- | --- | --- | --- | --- | |
| --- | --- | --- | --- | --- | --- | --- | --- | --- | --- |

| | | tr | [A1DU40](http://www.uniprot.org/uniprot/A1DU40) A1DU40_ARTSF | **60S acidic ribosomal protein P2 [Artemia sanfranciscana (Brine shrimp) (Artemia franciscana)]** | 111 AA | | --- | --- | --- | --- | | Score = 44.8 bits (98), Expect = 0.001  Identities = 12/13 (92%), Positives = 13/13 (100%)  Query: 1 EEEDDDMGFGLFD 13  EEED+DMGFGLFD  Sbjct: 99 EEEDEDMGFGLFD 111 | | | | | | --- | --- | --- | --- | --- | --- | --- | --- | --- | |
| --- | --- | --- | --- | --- | --- | --- | --- | --- | --- |

| | | tr | [A1KXI7](http://www.uniprot.org/uniprot/A1KXI7) A1KXI7_BLOTA | **Blo t Alt a 6 allergen [Blomia tropicalis (Mite)]** | 114 AA | | --- | --- | --- | --- | | Score = 44.3 bits (97), Expect = 0.001  Identities = 12/13 (92%), Positives = 13/13 (100%)  Query: 1 EEEDDDMGFGLFD 13  E+EDDDMGFGLFD  Sbjct: 102 EQEDDDMGFGLFD 114 | | | | | | --- | --- | --- | --- | --- | --- | --- | --- | --- | |
| --- | --- | --- | --- | --- | --- | --- | --- | --- | --- |

| | | sp | [Q9U3U0](http://www.uniprot.org/uniprot/Q9U3U0) RLA0_CERCA | **60S acidic ribosomal protein P0 (CcP0) [RpLP0] [Ceratitis capitata (Mediterranean fruit fly)]** | 317 AA | | --- | --- | --- | --- | | Score = 43.9 bits (96), Expect = 0.002  Identities = 12/12 (100%), Positives = 12/12 (100%)  Query: 2 EEDDDMGFGLFD 13  EEDDDMGFGLFD  Sbjct: 306 EEDDDMGFGLFD 317 | | | | | | --- | --- | --- | --- | --- | --- | --- | --- | --- | |
| --- | --- | --- | --- | --- | --- | --- | --- | --- | --- |

| | | tr | [Q4PMB4](http://www.uniprot.org/uniprot/Q4PMB4) Q4PMB4_IXOSC | **60S acidic ribosomal protein P0 [Ixodes scapularis (Black-legged tick) (Deer tick)]** | 319 AA | | --- | --- | --- | --- | | Score = 43.9 bits (96), Expect = 0.002  Identities = 12/13 (92%), Positives = 12/13 (92%)  Query: 1 EEEDDDMGFGLFD 13  EEEDDDMGF LFD  Sbjct: 307 EEEDDDMGFSLFD 319 | | | | | | --- | --- | --- | --- | --- | --- | --- | --- | --- | |
| --- | --- | --- | --- | --- | --- | --- | --- | --- | --- |

| | | tr | [C1PHC5](http://www.uniprot.org/uniprot/C1PHC5) C1PHC5_BABRO | **60S acidic ribosomal protein P0 (Fragment) [Babesia rodhaini]** | 311 AA | | --- | --- | --- | --- | | Score = 43.9 bits (96), Expect = 0.002  Identities = 12/13 (92%), Positives = 12/13 (92%)  Query: 1 EEEDDDMGFGLFD 13  EEEDDDMGF LFD  Sbjct: 299 EEEDDDMGFSLFD 311 | | | | | | --- | --- | --- | --- | --- | --- | --- | --- | --- | |
| --- | --- | --- | --- | --- | --- | --- | --- | --- | --- |

| | | tr | [B2ZRT0](http://www.uniprot.org/uniprot/B2ZRT0) B2ZRT0_9ACAR | **Ribosomal protein [LA-2] [Haemaphysalis qinghaiensis]** | 117 AA | | --- | --- | --- | --- | | Score = 43.9 bits (96), Expect = 0.002  Identities = 12/12 (100%), Positives = 12/12 (100%)  Query: 2 EEDDDMGFGLFD 13  EEDDDMGFGLFD  Sbjct: 106 EEDDDMGFGLFD 117 | | | | | | --- | --- | --- | --- | --- | --- | --- | --- | --- | |
| --- | --- | --- | --- | --- | --- | --- | --- | --- | --- |

| | | tr | [C4QYM0](http://www.uniprot.org/uniprot/C4QYM0) C4QYM0_PICPG | **60S acidic ribosomal protein P2-A [PAS_chr1-4_0490] [Pichia pastoris (strain GS115) (Yeast)]** | 118 AA | | --- | --- | --- | --- | | Score = 43.9 bits (96), Expect = 0.002  Identities = 12/12 (100%), Positives = 12/12 (100%)  Query: 2 EEDDDMGFGLFD 13  EEDDDMGFGLFD  Sbjct: 107 EEDDDMGFGLFD 118 | | | | | | --- | --- | --- | --- | --- | --- | --- | --- | --- | |
| --- | --- | --- | --- | --- | --- | --- | --- | --- | --- |

| | | tr | [A7TPM0](http://www.uniprot.org/uniprot/A7TPM0) A7TPM0_VANPO | **Putative uncharacterized protein [Kpol_1040p25] [Vanderwaltozyma polyspora (strain ATCC 22028 / DSM 70294) (Kluyveromyces polysporus)]** | 107 AA | | --- | --- | --- | --- | | Score = 43.9 bits (96), Expect = 0.002  Identities = 12/12 (100%), Positives = 12/12 (100%)  Query: 2 EEDDDMGFGLFD 13  EEDDDMGFGLFD  Sbjct: 96 EEDDDMGFGLFD 107 | | | | | | --- | --- | --- | --- | --- | --- | --- | --- | --- | |
| --- | --- | --- | --- | --- | --- | --- | --- | --- | --- |

| | | tr | [A7TF23](http://www.uniprot.org/uniprot/A7TF23) A7TF23_VANPO | **Putative uncharacterized protein [Kpol_2000p12] [Vanderwaltozyma polyspora (strain ATCC 22028 / DSM 70294) (Kluyveromyces polysporus)]** | 105 AA | | --- | --- | --- | --- | | Score = 43.9 bits (96), Expect = 0.002  Identities = 12/12 (100%), Positives = 12/12 (100%)  Query: 2 EEDDDMGFGLFD 13  EEDDDMGFGLFD  Sbjct: 94 EEDDDMGFGLFD 105 | | | | | | --- | --- | --- | --- | --- | --- | --- | --- | --- | |
| --- | --- | --- | --- | --- | --- | --- | --- | --- | --- |

| | | tr | [A7KMW4](http://www.uniprot.org/uniprot/A7KMW4) A7KMW4_9BASI | **60S acidic ribosomal protein P1 [Melampsora medusae f. sp. deltoidis]** | 109 AA | | --- | --- | --- | --- | | Score = 43.5 bits (95), Expect = 0.003  Identities = 12/13 (92%), Positives = 12/13 (92%)  Query: 1 EEEDDDMGFGLFD 13  EE DDDMGFGLFD  Sbjct: 97 EEXDDDMGFGLFD 109 | | | | | | --- | --- | --- | --- | --- | --- | --- | --- | --- | |
| --- | --- | --- | --- | --- | --- | --- | --- | --- | --- |

| | | sp | [Q06382](http://www.uniprot.org/uniprot/Q06382) RLA3_LEIIN | **60S acidic ribosomal protein P2-2 [LIP'] [Leishmania infantum]** | 111 AA | | --- | --- | --- | --- | | Score = 43.1 bits (94), Expect = 0.003  Identities = 12/13 (92%), Positives = 12/13 (92%)  Query: 1 EEEDDDMGFGLFD 13  EE DDDMGFGLFD  Sbjct: 99 EEADDDMGFGLFD 111 | | | | | | --- | --- | --- | --- | --- | --- | --- | --- | --- | |
| --- | --- | --- | --- | --- | --- | --- | --- | --- | --- |

| | | sp | [Q06383](http://www.uniprot.org/uniprot/Q06383) RLA2_LEIIN | **60S acidic ribosomal protein P2-1 [LIP] [Leishmania infantum]** | 106 AA | | --- | --- | --- | --- | | Score = 43.1 bits (94), Expect = 0.003  Identities = 12/13 (92%), Positives = 12/13 (92%)  Query: 1 EEEDDDMGFGLFD 13  EE DDDMGFGLFD  Sbjct: 94 EEADDDMGFGLFD 106 | | | | | | --- | --- | --- | --- | --- | --- | --- | --- | --- | |
| --- | --- | --- | --- | --- | --- | --- | --- | --- | --- |

| | | sp | [O43940](http://www.uniprot.org/uniprot/O43940) RLA2_LEIDO | **60S acidic ribosomal protein P2 (Acidic ribosomal protein 1) [ARP-1] [Leishmania donovani]** | 105 AA | | --- | --- | --- | --- | | Score = 43.1 bits (94), Expect = 0.003  Identities = 12/13 (92%), Positives = 12/13 (92%)  Query: 1 EEEDDDMGFGLFD 13  EE DDDMGFGLFD  Sbjct: 93 EEADDDMGFGLFD 105 | | | | | | --- | --- | --- | --- | --- | --- | --- | --- | --- | |
| --- | --- | --- | --- | --- | --- | --- | --- | --- | --- |

| | | sp | [O44010](http://www.uniprot.org/uniprot/O44010) RLA2_LEIBR | **60S acidic ribosomal protein P2 (Acidic ribosomal P2 beta protein) (P2B-protein) [Leishmania braziliensis]** | 105 AA | | --- | --- | --- | --- | | Score = 43.1 bits (94), Expect = 0.003  Identities = 12/13 (92%), Positives = 12/13 (92%)  Query: 1 EEEDDDMGFGLFD 13  EE DDDMGFGLFD  Sbjct: 93 EEADDDMGFGLFD 105 | | | | | | --- | --- | --- | --- | --- | --- | --- | --- | --- | |
| --- | --- | --- | --- | --- | --- | --- | --- | --- | --- |

| | | sp | [O61463](http://www.uniprot.org/uniprot/O61463) RLA2_CRYST | **60S acidic ribosomal protein P2 [Cryptochiton stelleri]** | 110 AA | | --- | --- | --- | --- | | Score = 43.1 bits (94), Expect = 0.003  Identities = 13/14 (92%), Positives = 13/14 (92%), Gaps = 1/14 (7%)  Query: 1 EEE-DDDMGFGLFD 13  EEE DDDMGFGLFD  Sbjct: 97 EEESDDDMGFGLFD 110 | | | | | | --- | --- | --- | --- | --- | --- | --- | --- | --- | |
| --- | --- | --- | --- | --- | --- | --- | --- | --- | --- |

| | | sp | [O01725](http://www.uniprot.org/uniprot/O01725) RLA2_BRAFL | **60S acidic ribosomal protein P2 [Branchiostoma floridae (Florida lancelet) (Amphioxus)]** | 116 AA | | --- | --- | --- | --- | | Score = 43.1 bits (94), Expect = 0.003  Identities = 13/14 (92%), Positives = 13/14 (92%), Gaps = 1/14 (7%)  Query: 1 EEE-DDDMGFGLFD 13  EEE DDDMGFGLFD  Sbjct: 103 EEESDDDMGFGLFD 116 | | | | | | --- | --- | --- | --- | --- | --- | --- | --- | --- | |
| --- | --- | --- | --- | --- | --- | --- | --- | --- | --- |

| | | sp | [O46313](http://www.uniprot.org/uniprot/O46313) RLA1_LEIPE | **60S acidic ribosomal protein P1 [Leishmania peruviana]** | 107 AA | | --- | --- | --- | --- | | Score = 43.1 bits (94), Expect = 0.003  Identities = 13/14 (92%), Positives = 13/14 (92%), Gaps = 1/14 (7%)  Query: 1 EEE-DDDMGFGLFD 13  EEE DDDMGFGLFD  Sbjct: 94 EEEGDDDMGFGLFD 107 | | | | | | --- | --- | --- | --- | --- | --- | --- | --- | --- | |
| --- | --- | --- | --- | --- | --- | --- | --- | --- | --- |

| | | sp | [P05317](http://www.uniprot.org/uniprot/P05317) RLA0_YEAST | **60S acidic ribosomal protein P0 (A0) (L10E) [RPP0] [Saccharomyces cerevisiae (Baker's yeast)]** | 312 AA | | --- | --- | --- | --- | | Score = 43.1 bits (94), Expect = 0.003  Identities = 13/14 (92%), Positives = 13/14 (92%), Gaps = 1/14 (7%)  Query: 1 EEE-DDDMGFGLFD 13  EEE DDDMGFGLFD  Sbjct: 299 EEESDDDMGFGLFD 312 | | | | | | --- | --- | --- | --- | --- | --- | --- | --- | --- | |
| --- | --- | --- | --- | --- | --- | --- | --- | --- | --- |

| | | tr | [Q9N6F4](http://www.uniprot.org/uniprot/Q9N6F4) Q9N6F4_LEIMA | **60S acidic ribosomal protein P2, putative [LMJ_0200] [Leishmania major]** | 111 AA | | --- | --- | --- | --- | | Score = 43.1 bits (94), Expect = 0.003  Identities = 13/14 (92%), Positives = 13/14 (92%), Gaps = 1/14 (7%)  Query: 1 EEE-DDDMGFGLFD 13  EEE DDDMGFGLFD  Sbjct: 98 EEEGDDDMGFGLFD 111 | | | | | | --- | --- | --- | --- | --- | --- | --- | --- | --- | |
| --- | --- | --- | --- | --- | --- | --- | --- | --- | --- |

| | | tr | [Q8ISP6](http://www.uniprot.org/uniprot/Q8ISP6) Q8ISP6_BRABE | **Ribosomal protein P1 [Branchiostoma belcheri (Amphioxus)]** | 113 AA | | --- | --- | --- | --- | | Score = 43.1 bits (94), Expect = 0.003  Identities = 13/14 (92%), Positives = 13/14 (92%), Gaps = 1/14 (7%)  Query: 1 EEE-DDDMGFGLFD 13  EEE DDDMGFGLFD  Sbjct: 100 EEESDDDMGFGLFD 113 | | | | | | --- | --- | --- | --- | --- | --- | --- | --- | --- | |
| --- | --- | --- | --- | --- | --- | --- | --- | --- | --- |

| | | tr | [Q8I867](http://www.uniprot.org/uniprot/Q8I867) Q8I867_LEIDO | **Ribosomal protein P1-like protein [Leishmania donovani]** | 111 AA | | --- | --- | --- | --- | | Score = 43.1 bits (94), Expect = 0.003  Identities = 13/14 (92%), Positives = 13/14 (92%), Gaps = 1/14 (7%)  Query: 1 EEE-DDDMGFGLFD 13  EEE DDDMGFGLFD  Sbjct: 98 EEEGDDDMGFGLFD 111 | | | | | | --- | --- | --- | --- | --- | --- | --- | --- | --- | |
| --- | --- | --- | --- | --- | --- | --- | --- | --- | --- |

| | | tr | [Q70YZ3](http://www.uniprot.org/uniprot/Q70YZ3) Q70YZ3_BOMMO | **Ribosomal P0 protein [Bombyx mori (Silk moth)]** | 316 AA | | --- | --- | --- | --- | | Score = 43.1 bits (94), Expect = 0.003  Identities = 13/14 (92%), Positives = 13/14 (92%), Gaps = 1/14 (7%)  Query: 1 EEE-DDDMGFGLFD 13  EEE DDDMGFGLFD  Sbjct: 303 EEESDDDMGFGLFD 316 | | | | | | --- | --- | --- | --- | --- | --- | --- | --- | --- | |
| --- | --- | --- | --- | --- | --- | --- | --- | --- | --- |

| | | tr | [Q66VD2](http://www.uniprot.org/uniprot/Q66VD2) Q66VD2_LEIDO | **Acidic ribosomal protein [Leishmania donovani]** | 105 AA | | --- | --- | --- | --- | | Score = 43.1 bits (94), Expect = 0.003  Identities = 12/13 (92%), Positives = 12/13 (92%)  Query: 1 EEEDDDMGFGLFD 13  EE DDDMGFGLFD  Sbjct: 93 EEADDDMGFGLFD 105 | | | | | | --- | --- | --- | --- | --- | --- | --- | --- | --- | |
| --- | --- | --- | --- | --- | --- | --- | --- | --- | --- |

| | | tr | [Q5UAU1](http://www.uniprot.org/uniprot/Q5UAU1) Q5UAU1_BOMMO | **Ribosomal protein P0 [RpP0] [Bombyx mori (Silk moth)]** | 316 AA | | --- | --- | --- | --- | | Score = 43.1 bits (94), Expect = 0.003  Identities = 13/14 (92%), Positives = 13/14 (92%), Gaps = 1/14 (7%)  Query: 1 EEE-DDDMGFGLFD 13  EEE DDDMGFGLFD  Sbjct: 303 EEESDDDMGFGLFD 316 | | | | | | --- | --- | --- | --- | --- | --- | --- | --- | --- | |
| --- | --- | --- | --- | --- | --- | --- | --- | --- | --- |

| | | tr | [Q4QF62](http://www.uniprot.org/uniprot/Q4QF62) Q4QF62_LEIMA | **60S acidic ribosomal protein P2 [LmjF15.1203] [Leishmania major]** | 111 AA | | --- | --- | --- | --- | | Score = 43.1 bits (94), Expect = 0.003  Identities = 12/13 (92%), Positives = 12/13 (92%)  Query: 1 EEEDDDMGFGLFD 13  EE DDDMGFGLFD  Sbjct: 99 EEADDDMGFGLFD 111 | | | | | | --- | --- | --- | --- | --- | --- | --- | --- | --- | |
| --- | --- | --- | --- | --- | --- | --- | --- | --- | --- |

| | | tr | [Q4Q6R6](http://www.uniprot.org/uniprot/Q4Q6R6) Q4Q6R6_LEIMA | **60S acidic ribosomal protein P2, putative [LmjF30.3720] [Leishmania major]** | 106 AA | | --- | --- | --- | --- | | Score = 43.1 bits (94), Expect = 0.003  Identities = 12/13 (92%), Positives = 12/13 (92%)  Query: 1 EEEDDDMGFGLFD 13  EE DDDMGFGLFD  Sbjct: 94 EEADDDMGFGLFD 106 | | | | | | --- | --- | --- | --- | --- | --- | --- | --- | --- | |
| --- | --- | --- | --- | --- | --- | --- | --- | --- | --- |

| | | tr | [Q4Q6R5](http://www.uniprot.org/uniprot/Q4Q6R5) Q4Q6R5_LEIMA | **60S acidic ribosomal protein P2, putative [LmjF30.3730] [Leishmania major]** | 125 AA | | --- | --- | --- | --- | | Score = 43.1 bits (94), Expect = 0.003  Identities = 12/13 (92%), Positives = 12/13 (92%)  Query: 1 EEEDDDMGFGLFD 13  EE DDDMGFGLFD  Sbjct: 113 EEADDDMGFGLFD 125 | | | | | | --- | --- | --- | --- | --- | --- | --- | --- | --- | |
| --- | --- | --- | --- | --- | --- | --- | --- | --- | --- |

| | | tr | [Q4KTI8](http://www.uniprot.org/uniprot/Q4KTI8) Q4KTI8_SUBDO | **P1 [Suberites domuncula (Sponge)]** | 108 AA | | --- | --- | --- | --- | | Score = 43.1 bits (94), Expect = 0.003  Identities = 13/14 (92%), Positives = 13/14 (92%), Gaps = 1/14 (7%)  Query: 1 EEE-DDDMGFGLFD 13  EEE DDDMGFGLFD  Sbjct: 95 EEESDDDMGFGLFD 108 | | | | | | --- | --- | --- | --- | --- | --- | --- | --- | --- | |
| --- | --- | --- | --- | --- | --- | --- | --- | --- | --- |

| | | tr | [Q4KTI7](http://www.uniprot.org/uniprot/Q4KTI7) Q4KTI7_SUBDO | **P2 [Suberites domuncula (Sponge)]** | 111 AA | | --- | --- | --- | --- | | Score = 43.1 bits (94), Expect = 0.003  Identities = 13/14 (92%), Positives = 13/14 (92%), Gaps = 1/14 (7%)  Query: 1 EEE-DDDMGFGLFD 13  EEE DDDMGFGLFD  Sbjct: 98 EEESDDDMGFGLFD 111 | | | | | | --- | --- | --- | --- | --- | --- | --- | --- | --- | |
| --- | --- | --- | --- | --- | --- | --- | --- | --- | --- |

| | | tr | [Q4KTH7](http://www.uniprot.org/uniprot/Q4KTH7) Q4KTH7_SUBDO | **L10e/P0 [Suberites domuncula (Sponge)]** | 313 AA | | --- | --- | --- | --- | | Score = 43.1 bits (94), Expect = 0.003  Identities = 13/14 (92%), Positives = 13/14 (92%), Gaps = 1/14 (7%)  Query: 1 EEE-DDDMGFGLFD 13  EEE DDDMGFGLFD  Sbjct: 300 EEESDDDMGFGLFD 313 | | | | | | --- | --- | --- | --- | --- | --- | --- | --- | --- | |
| --- | --- | --- | --- | --- | --- | --- | --- | --- | --- |

| | | tr | [Q1HCM5](http://www.uniprot.org/uniprot/Q1HCM5) Q1HCM5_PECGU | **Ribosomal protein P1 [Pectinaria gouldii (Trumpet worm) (Ice-cream cone worm)]** | 111 AA | | --- | --- | --- | --- | | Score = 43.1 bits (94), Expect = 0.003  Identities = 13/14 (92%), Positives = 13/14 (92%), Gaps = 1/14 (7%)  Query: 1 EEE-DDDMGFGLFD 13  EEE DDDMGFGLFD  Sbjct: 98 EEESDDDMGFGLFD 111 | | | | | | --- | --- | --- | --- | --- | --- | --- | --- | --- | |
| --- | --- | --- | --- | --- | --- | --- | --- | --- | --- |

| | | tr | [C3ZD79](http://www.uniprot.org/uniprot/C3ZD79) C3ZD79_BRAFL | **Putative uncharacterized protein [BRAFLDRAFT_206448] [Branchiostoma floridae (Florida lancelet) (Amphioxus)]** | 109 AA | | --- | --- | --- | --- | | Score = 43.1 bits (94), Expect = 0.003  Identities = 13/14 (92%), Positives = 13/14 (92%), Gaps = 1/14 (7%)  Query: 1 EEE-DDDMGFGLFD 13  EEE DDDMGFGLFD  Sbjct: 96 EEESDDDMGFGLFD 109 | | | | | | --- | --- | --- | --- | --- | --- | --- | --- | --- | |
| --- | --- | --- | --- | --- | --- | --- | --- | --- | --- |

| | | tr | [C3Z9A6](http://www.uniprot.org/uniprot/C3Z9A6) C3Z9A6_BRAFL | **Putative uncharacterized protein [BRAFLDRAFT_124423] [Branchiostoma floridae (Florida lancelet) (Amphioxus)]** | 115 AA | | --- | --- | --- | --- | | Score = 43.1 bits (94), Expect = 0.003  Identities = 13/14 (92%), Positives = 13/14 (92%), Gaps = 1/14 (7%)  Query: 1 EEE-DDDMGFGLFD 13  EEE DDDMGFGLFD  Sbjct: 102 EEESDDDMGFGLFD 115 | | | | | | --- | --- | --- | --- | --- | --- | --- | --- | --- | |
| --- | --- | --- | --- | --- | --- | --- | --- | --- | --- |

| | | tr | [C3Z0M5](http://www.uniprot.org/uniprot/C3Z0M5) C3Z0M5_BRAFL | **Putative uncharacterized protein [BRAFLDRAFT_97880] [Branchiostoma floridae (Florida lancelet) (Amphioxus)]** | 314 AA | | --- | --- | --- | --- | | Score = 43.1 bits (94), Expect = 0.003  Identities = 13/14 (92%), Positives = 13/14 (92%), Gaps = 1/14 (7%)  Query: 1 EEE-DDDMGFGLFD 13  EEE DDDMGFGLFD  Sbjct: 301 EEESDDDMGFGLFD 314 | | | | | | --- | --- | --- | --- | --- | --- | --- | --- | --- | |
| --- | --- | --- | --- | --- | --- | --- | --- | --- | --- |

| | | tr | [C1BV18](http://www.uniprot.org/uniprot/C1BV18) C1BV18_9MAXI | **60S acidic ribosomal protein P2 [RLA2] [Lepeophtheirus salmonis (salmon louse)]** | 110 AA | | --- | --- | --- | --- | | Score = 43.1 bits (94), Expect = 0.003  Identities = 13/14 (92%), Positives = 13/14 (92%), Gaps = 1/14 (7%)  Query: 1 EEE-DDDMGFGLFD 13  EEE DDDMGFGLFD  Sbjct: 97 EEESDDDMGFGLFD 110 | | | | | | --- | --- | --- | --- | --- | --- | --- | --- | --- | |
| --- | --- | --- | --- | --- | --- | --- | --- | --- | --- |

| | | tr | [C1BTD7](http://www.uniprot.org/uniprot/C1BTD7) C1BTD7_9MAXI | **60S acidic ribosomal protein P0 [RLA0] [Lepeophtheirus salmonis (salmon louse)]** | 314 AA | | --- | --- | --- | --- | | Score = 43.1 bits (94), Expect = 0.003  Identities = 13/14 (92%), Positives = 13/14 (92%), Gaps = 1/14 (7%)  Query: 1 EEE-DDDMGFGLFD 13  EEE DDDMGFGLFD  Sbjct: 301 EEESDDDMGFGLFD 314 | | | | | | --- | --- | --- | --- | --- | --- | --- | --- | --- | |
| --- | --- | --- | --- | --- | --- | --- | --- | --- | --- |

| | | tr | [B6ZCB2](http://www.uniprot.org/uniprot/B6ZCB2) B6ZCB2_MYTGA | **Ribosomal protein [p0] [Mytilus galloprovincialis (Mediterranean mussel)]** | 315 AA | | --- | --- | --- | --- | | Score = 43.1 bits (94), Expect = 0.003  Identities = 13/14 (92%), Positives = 13/14 (92%), Gaps = 1/14 (7%)  Query: 1 EEE-DDDMGFGLFD 13  EEE DDDMGFGLFD  Sbjct: 302 EEESDDDMGFGLFD 315 | | | | | | --- | --- | --- | --- | --- | --- | --- | --- | --- | |
| --- | --- | --- | --- | --- | --- | --- | --- | --- | --- |

| | | tr | [B4XPB7](http://www.uniprot.org/uniprot/B4XPB7) B4XPB7_9CNID | **60S acidic ribosomal phosphoprotein P0 [36B4] [Stylophora pistillata]** | 317 AA | | --- | --- | --- | --- | | Score = 43.1 bits (94), Expect = 0.003  Identities = 13/14 (92%), Positives = 13/14 (92%), Gaps = 1/14 (7%)  Query: 1 EEE-DDDMGFGLFD 13  EEE DDDMGFGLFD  Sbjct: 304 EEESDDDMGFGLFD 317 | | | | | | --- | --- | --- | --- | --- | --- | --- | --- | --- | |
| --- | --- | --- | --- | --- | --- | --- | --- | --- | --- |

| | | tr | [B3S429](http://www.uniprot.org/uniprot/B3S429) B3S429_TRIAD | **Putative uncharacterized protein [TRIADDRAFT_63360] [Trichoplax adhaerens]** | 314 AA | | --- | --- | --- | --- | | Score = 43.1 bits (94), Expect = 0.003  Identities = 13/14 (92%), Positives = 13/14 (92%), Gaps = 1/14 (7%)  Query: 1 EEE-DDDMGFGLFD 13  EEE DDDMGFGLFD  Sbjct: 301 EEESDDDMGFGLFD 314 | | | | | | --- | --- | --- | --- | --- | --- | --- | --- | --- | |
| --- | --- | --- | --- | --- | --- | --- | --- | --- | --- |

| | | tr | [B3S1Z8](http://www.uniprot.org/uniprot/B3S1Z8) B3S1Z8_TRIAD | **Putative uncharacterized protein [TRIADDRAFT_63121] [Trichoplax adhaerens]** | 110 AA | | --- | --- | --- | --- | | Score = 43.1 bits (94), Expect = 0.003  Identities = 13/14 (92%), Positives = 13/14 (92%), Gaps = 1/14 (7%)  Query: 1 EEE-DDDMGFGLFD 13  EEE DDDMGFGLFD  Sbjct: 97 EEESDDDMGFGLFD 110 | | | | | | --- | --- | --- | --- | --- | --- | --- | --- | --- | |
| --- | --- | --- | --- | --- | --- | --- | --- | --- | --- |

| | | tr | [B3S1V3](http://www.uniprot.org/uniprot/B3S1V3) B3S1V3_TRIAD | **Putative uncharacterized protein [TRIADDRAFT_37976] [Trichoplax adhaerens]** | 112 AA | | --- | --- | --- | --- | | Score = 43.1 bits (94), Expect = 0.003  Identities = 13/14 (92%), Positives = 13/14 (92%), Gaps = 1/14 (7%)  Query: 1 EEE-DDDMGFGLFD 13  EEE DDDMGFGLFD  Sbjct: 99 EEESDDDMGFGLFD 112 | | | | | | --- | --- | --- | --- | --- | --- | --- | --- | --- | |
| --- | --- | --- | --- | --- | --- | --- | --- | --- | --- |

| | | tr | [B3GQX2](http://www.uniprot.org/uniprot/B3GQX2) B3GQX2_ASTPE | **60S acidic ribosomal protein P2 [Asterina pectinifera (Starfish) (Patiria pectinifera)]** | 112 AA | | --- | --- | --- | --- | | Score = 43.1 bits (94), Expect = 0.003  Identities = 13/14 (92%), Positives = 13/14 (92%), Gaps = 1/14 (7%)  Query: 1 EEE-DDDMGFGLFD 13  EEE DDDMGFGLFD  Sbjct: 99 EEESDDDMGFGLFD 112 | | | | | | --- | --- | --- | --- | --- | --- | --- | --- | --- | |
| --- | --- | --- | --- | --- | --- | --- | --- | --- | --- |

| | | tr | [B0Z9P2](http://www.uniprot.org/uniprot/B0Z9P2) B0Z9P2_9BILA | **Ribosomal protein rplp2 [Lineus viridis]** | 111 AA | | --- | --- | --- | --- | | Score = 43.1 bits (94), Expect = 0.003  Identities = 13/14 (92%), Positives = 13/14 (92%), Gaps = 1/14 (7%)  Query: 1 EEE-DDDMGFGLFD 13  EEE DDDMGFGLFD  Sbjct: 98 EEESDDDMGFGLFD 111 | | | | | | --- | --- | --- | --- | --- | --- | --- | --- | --- | |
| --- | --- | --- | --- | --- | --- | --- | --- | --- | --- |

| | | tr | [B0Z9P1](http://www.uniprot.org/uniprot/B0Z9P1) B0Z9P1_9BILA | **Ribosomal protein rplp1 [Lineus viridis]** | 111 AA | | --- | --- | --- | --- | | Score = 43.1 bits (94), Expect = 0.003  Identities = 13/14 (92%), Positives = 13/14 (92%), Gaps = 1/14 (7%)  Query: 1 EEE-DDDMGFGLFD 13  EEE DDDMGFGLFD  Sbjct: 98 EEESDDDMGFGLFD 111 | | | | | | --- | --- | --- | --- | --- | --- | --- | --- | --- | |
| --- | --- | --- | --- | --- | --- | --- | --- | --- | --- |

| | | tr | [B0Z9P0](http://www.uniprot.org/uniprot/B0Z9P0) B0Z9P0_9BILA | **Ribosomal protein rplp0 [Lineus viridis]** | 315 AA | | --- | --- | --- | --- | | Score = 43.1 bits (94), Expect = 0.003  Identities = 13/14 (92%), Positives = 13/14 (92%), Gaps = 1/14 (7%)  Query: 1 EEE-DDDMGFGLFD 13  EEE DDDMGFGLFD  Sbjct: 302 EEESDDDMGFGLFD 315 | | | | | | --- | --- | --- | --- | --- | --- | --- | --- | --- | |
| --- | --- | --- | --- | --- | --- | --- | --- | --- | --- |

| | | tr | [A7SQ36](http://www.uniprot.org/uniprot/A7SQ36) A7SQ36_NEMVE | **Predicted protein [v1g237425] [Nematostella vectensis (Starlet sea anemone)]** | 313 AA | | --- | --- | --- | --- | | Score = 43.1 bits (94), Expect = 0.003  Identities = 13/14 (92%), Positives = 13/14 (92%), Gaps = 1/14 (7%)  Query: 1 EEE-DDDMGFGLFD 13  EEE DDDMGFGLFD  Sbjct: 300 EEESDDDMGFGLFD 313 | | | | | | --- | --- | --- | --- | --- | --- | --- | --- | --- | |
| --- | --- | --- | --- | --- | --- | --- | --- | --- | --- |

| | | tr | [A7S837](http://www.uniprot.org/uniprot/A7S837) A7S837_NEMVE | **Predicted protein [v1g186829] [Nematostella vectensis (Starlet sea anemone)]** | 110 AA | | --- | --- | --- | --- | | Score = 43.1 bits (94), Expect = 0.003  Identities = 13/14 (92%), Positives = 13/14 (92%), Gaps = 1/14 (7%)  Query: 1 EEE-DDDMGFGLFD 13  EEE DDDMGFGLFD  Sbjct: 97 EEESDDDMGFGLFD 110 | | | | | | --- | --- | --- | --- | --- | --- | --- | --- | --- | |
| --- | --- | --- | --- | --- | --- | --- | --- | --- | --- |

| | | tr | [A7RLY2](http://www.uniprot.org/uniprot/A7RLY2) A7RLY2_NEMVE | **Predicted protein [v1g160310] [Nematostella vectensis (Starlet sea anemone)]** | 113 AA | | --- | --- | --- | --- | | Score = 43.1 bits (94), Expect = 0.003  Identities = 13/14 (92%), Positives = 13/14 (92%), Gaps = 1/14 (7%)  Query: 1 EEE-DDDMGFGLFD 13  EEE DDDMGFGLFD  Sbjct: 100 EEESDDDMGFGLFD 113 | | | | | | --- | --- | --- | --- | --- | --- | --- | --- | --- | |
| --- | --- | --- | --- | --- | --- | --- | --- | --- | --- |

| | | tr | [A4I600](http://www.uniprot.org/uniprot/A4I600) A4I600_LEIIN | **60S acidic ribosomal protein P2, putative [LinJ30.3580] [Leishmania infantum]** | 105 AA | | --- | --- | --- | --- | | Score = 43.1 bits (94), Expect = 0.003  Identities = 12/13 (92%), Positives = 12/13 (92%)  Query: 1 EEEDDDMGFGLFD 13  EE DDDMGFGLFD  Sbjct: 93 EEADDDMGFGLFD 105 | | | | | | --- | --- | --- | --- | --- | --- | --- | --- | --- | |
| --- | --- | --- | --- | --- | --- | --- | --- | --- | --- |

| | | tr | [A4I5Z9](http://www.uniprot.org/uniprot/A4I5Z9) A4I5Z9_LEIIN | **60S acidic ribosomal protein P2, putative [LinJ30.3570] [Leishmania infantum]** | 105 AA | | --- | --- | --- | --- | | Score = 43.1 bits (94), Expect = 0.003  Identities = 12/13 (92%), Positives = 12/13 (92%)  Query: 1 EEEDDDMGFGLFD 13  EE DDDMGFGLFD  Sbjct: 93 EEADDDMGFGLFD 105 | | | | | | --- | --- | --- | --- | --- | --- | --- | --- | --- | |
| --- | --- | --- | --- | --- | --- | --- | --- | --- | --- |

| | | tr | [A4HRV4](http://www.uniprot.org/uniprot/A4HRV4) A4HRV4_LEIIN | **60S acidic ribosomal protein P2, putative [LinJ03.0280] [Leishmania infantum]** | 111 AA | | --- | --- | --- | --- | | Score = 43.1 bits (94), Expect = 0.003  Identities = 13/14 (92%), Positives = 13/14 (92%), Gaps = 1/14 (7%)  Query: 1 EEE-DDDMGFGLFD 13  EEE DDDMGFGLFD  Sbjct: 98 EEEGDDDMGFGLFD 111 | | | | | | --- | --- | --- | --- | --- | --- | --- | --- | --- | |
| --- | --- | --- | --- | --- | --- | --- | --- | --- | --- |

| | | tr | [A4HIV9](http://www.uniprot.org/uniprot/A4HIV9) A4HIV9_LEIBR | **60S acidic ribosomal protein P2, putative [LbrM30_V2.3760] [Leishmania braziliensis]** | 105 AA | | --- | --- | --- | --- | | Score = 43.1 bits (94), Expect = 0.003  Identities = 12/13 (92%), Positives = 12/13 (92%)  Query: 1 EEEDDDMGFGLFD 13  EE DDDMGFGLFD  Sbjct: 93 EEADDDMGFGLFD 105 | | | | | | --- | --- | --- | --- | --- | --- | --- | --- | --- | |
| --- | --- | --- | --- | --- | --- | --- | --- | --- | --- |

| | | tr | [A4H884](http://www.uniprot.org/uniprot/A4H884) A4H884_LEIBR | **60S acidic ribosomal protein P2 [LbrM15_V2.1150] [Leishmania braziliensis]** | 112 AA | | --- | --- | --- | --- | | Score = 43.1 bits (94), Expect = 0.003  Identities = 12/13 (92%), Positives = 12/13 (92%)  Query: 1 EEEDDDMGFGLFD 13  EE DDDMGFGLFD  Sbjct: 100 EEADDDMGFGLFD 112 | | | | | | --- | --- | --- | --- | --- | --- | --- | --- | --- | |
| --- | --- | --- | --- | --- | --- | --- | --- | --- | --- |

| | | tr | [A4H3L1](http://www.uniprot.org/uniprot/A4H3L1) A4H3L1_LEIBR | **60S acidic ribosomal protein P2, putative [LbrM03_V2.0400] [Leishmania braziliensis]** | 107 AA | | --- | --- | --- | --- | | Score = 43.1 bits (94), Expect = 0.003  Identities = 13/14 (92%), Positives = 13/14 (92%), Gaps = 1/14 (7%)  Query: 1 EEE-DDDMGFGLFD 13  EEE DDDMGFGLFD  Sbjct: 94 EEEGDDDMGFGLFD 107 | | | | | | --- | --- | --- | --- | --- | --- | --- | --- | --- | |
| --- | --- | --- | --- | --- | --- | --- | --- | --- | --- |

| | | tr | [A4H3L0](http://www.uniprot.org/uniprot/A4H3L0) A4H3L0_LEIBR | **60S acidic ribosomal protein P2, putative [LbrM03_V2.0390] [Leishmania braziliensis]** | 93 AA | | --- | --- | --- | --- | | Score = 43.1 bits (94), Expect = 0.003  Identities = 13/14 (92%), Positives = 13/14 (92%), Gaps = 1/14 (7%)  Query: 1 EEE-DDDMGFGLFD 13  EEE DDDMGFGLFD  Sbjct: 80 EEEGDDDMGFGLFD 93 | | | | | | --- | --- | --- | --- | --- | --- | --- | --- | --- | |
| --- | --- | --- | --- | --- | --- | --- | --- | --- | --- |

| | | tr | [A2I3Y7](http://www.uniprot.org/uniprot/A2I3Y7) A2I3Y7_MACHI | **60S acidic ribosomal protein P1-like protein [Maconellicoccus hirsutus (Pink hibiscus mealybug)]** | 113 AA | | --- | --- | --- | --- | | Score = 43.1 bits (94), Expect = 0.003  Identities = 13/14 (92%), Positives = 13/14 (92%), Gaps = 1/14 (7%)  Query: 1 EEE-DDDMGFGLFD 13  EEE DDDMGFGLFD  Sbjct: 100 EEESDDDMGFGLFD 113 | | | | | | --- | --- | --- | --- | --- | --- | --- | --- | --- | |
| --- | --- | --- | --- | --- | --- | --- | --- | --- | --- |

| | | tr | [Q75CU7](http://www.uniprot.org/uniprot/Q75CU7) Q75CU7_ASHGO | **ACL178Cp [ACL178C] [Ashbya gossypii (Yeast) (Eremothecium gossypii)]** | 309 AA | | --- | --- | --- | --- | | Score = 43.1 bits (94), Expect = 0.003  Identities = 13/14 (92%), Positives = 13/14 (92%), Gaps = 1/14 (7%)  Query: 1 EE-EDDDMGFGLFD 13  EE EDDDMGFGLFD  Sbjct: 296 EESEDDDMGFGLFD 309 | | | | | | --- | --- | --- | --- | --- | --- | --- | --- | --- | |
| --- | --- | --- | --- | --- | --- | --- | --- | --- | --- |

| | | tr | [Q6CW89](http://www.uniprot.org/uniprot/Q6CW89) Q6CW89_KLULA | **KLLA0B05918p [KLLA0B05918g] [Kluyveromyces lactis (Yeast) (Candida sphaerica)]** | 311 AA | | --- | --- | --- | --- | | Score = 43.1 bits (94), Expect = 0.003  Identities = 13/14 (92%), Positives = 13/14 (92%), Gaps = 1/14 (7%)  Query: 1 EEE-DDDMGFGLFD 13  EEE DDDMGFGLFD  Sbjct: 298 EEESDDDMGFGLFD 311 | | | | | | --- | --- | --- | --- | --- | --- | --- | --- | --- | |
| --- | --- | --- | --- | --- | --- | --- | --- | --- | --- |

Database: UniProtKB; Posted date: Sep 1, 2009 2:21 AM

Number of letters in database: 3,150,505,678

Number of sequences in database: 9,670,896

Lambda K H

0.321 0.292 1.72

Gapped Lambda K H

0.294 0.110 0.610

Matrix: PAM30

Gap Penalties: Existence: 9, Extension: 1

Number of Sequences: 9670896

Number of Hits to DB: 32,910,218

Number of extensions: 133621

Number of successful extensions: 12406

Number of sequences better than 10.0: 790

Number of HSP's gapped: 12418

Number of HSP's successfully gapped: 792

Length of query: 13

Length of database: 3,150,505,678

Length adjustment: 3

Effective length of query: 10

Effective length of database: 3,121,492,990

Effective search space: 31214929900

Effective search space used: 31214929900

Neighboring words threshold: 16

Window for multiple hits: 15

X1: 16 ( 7.4 bits)

X2: 35 (14.8 bits)

X3: 58 (24.6 bits)

S1: 43 (21.7 bits)

S2: 67 (31.6 bits)

Wallclock time: 19 seconds

| [**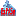ExPASy Home page**](http://www.expasy.ch/) | [**Site Map**](http://www.expasy.ch/sitemap.html) | [**Search ExPASy**](http://www.expasy.ch/ExpasyHunt/) | [**Contact us**](http://www.expasy.ch/contact.html) | [**Swiss-Prot**](http://www.expasy.ch/sprot/) | [**Proteomics tools**](http://www.expasy.ch/tools/) |
| --- | --- | --- | --- | --- | --- |
