# Supplementary material for: Searching for New Clues about the Molecular Cause of Endomyocardial Fibrosis by Way of In Silico Proteomics and Analytical Chemistry
Source: PLoS One. 2009 Oct 12;4(10):e7420. doi: 10.1371/journal.pone.0007420 (PMC2757908; doi:10.1371/journal.pone.0007420)

| [**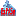ExPASy Home page**](http://www.expasy.ch/) | [**Site Map**](http://www.expasy.ch/sitemap.html) | [**Search ExPASy**](http://www.expasy.ch/ExpasyHunt/) | [**Contact us**](http://www.expasy.ch/contact.html) | [**Proteomics tools**](http://www.expasy.ch/tools/) | [**Swiss-Prot**](http://www.expasy.ch/sprot/) |
| --- | --- | --- | --- | --- | --- |

**User-provided sequence:** 10 EEEDDDMGFG LFD

**Number of amino acids:** 13

**Molecular weight:** 1518.5

**Theoretical pI:** 3.38

Top of Form

**Amino acid composition:** 
Ala (A) 0 0.0%

Arg (R) 0 0.0%

Asn (N) 0 0.0%

Asp (D) 4 30.8%

Cys (C) 0 0.0%

Gln (Q) 0 0.0%

Glu (E) 3 23.1%

Gly (G) 2 15.4%

His (H) 0 0.0%

Ile (I) 0 0.0%

Leu (L) 1 7.7%

Lys (K) 0 0.0%

Met (M) 1 7.7%

Phe (F) 2 15.4%

Pro (P) 0 0.0%

Ser (S) 0 0.0%

Thr (T) 0 0.0%

Trp (W) 0 0.0%

Tyr (Y) 0 0.0%

Val (V) 0 0.0%

Pyl (O) 0 0.0%

Sec (U) 0 0.0%

(B) 0 0.0%

(Z) 0 0.0%

(X) 0 0.0%

Bottom of Form

**Total number of negatively charged residues (Asp + Glu):** 7

**Total number of positively charged residues (Arg + Lys):** 0

**Atomic composition:**

Carbon C 64

Hydrogen H 87

Nitrogen N 13

Oxygen O 28

Sulfur S 1

**Formula:** C_64_H_87_N_13_O_28_S_1_

**Total number of atoms:** 193

**Extinction coefficients:**As there are no Trp, Tyr or Cys in the region considered, your protein should not be visible by UV spectrophotometry.

**Estimated half-life:**The N-terminal of the sequence considered is E (Glu).The estimated half-life is:

- 1 hour(s) (mammalian reticulocytes, in vitro).
- 30 min (yeast, in vivo).
- >10 hours (Escherichia coli, in vivo).

**Instability index:** The instability index (II) is computed to be 83.69. This classifies the protein as unstable.

**Aliphatic index:** 30.00

**Grand average of hydropathicity (GRAVY):** -1.077

| [**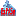ExPASy Home page**](http://www.expasy.ch/) | [**Site Map**](http://www.expasy.ch/sitemap.html) | [**Search ExPASy**](http://www.expasy.ch/ExpasyHunt/) | [**Contact us**](http://www.expasy.ch/contact.html) | [**Proteomics tools**](http://www.expasy.ch/tools/) | [**Swiss-Prot**](http://www.expasy.ch/sprot/) |
| --- | --- | --- | --- | --- | --- |


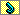

Supplement: File S3 — Showing the Biophysical profiles of the acidic C-terminus of the T.cruzi ribosomal P protein TcP0. The file provides details of the biophysical profiles, including chemical structure, amino acid composition, Theoretical PI, Instability index, Extinction coefficient Aliphatic Index and Graavy of the C-terminus peptide of TcP0 (EDDDDDFGMGALF). The data was generated by the Expasy software Protparam [25] (0.24 MB DOC) [file pone.0007420.s004.doc]
