# Supplementary material for: Searching for New Clues about the Molecular Cause of Endomyocardial Fibrosis by Way of In Silico Proteomics and Analytical Chemistry
Source: PLoS One. 2009 Oct 12;4(10):e7420. doi: 10.1371/journal.pone.0007420 (PMC2757908; doi:10.1371/journal.pone.0007420)

| [**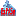ExPASy Home page**](http://www.expasy.ch/) | [**Site Map**](http://www.expasy.ch/sitemap.html) | [**Search ExPASy**](http://www.expasy.ch/ExpasyHunt/) | [**Contact us**](http://www.expasy.ch/contact.html) | [**Proteomics tools**](http://www.expasy.ch/tools/) | [**Swiss-Prot**](http://www.expasy.ch/sprot/) |
| --- | --- | --- | --- | --- | --- |

**
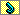
ProtParam**

**User-provided sequence:** 10 EDDDDDFGMG ALF

**Number of amino acids:** 13

**Molecular weight:** 1446.4

**Theoretical pI:** 3.21

Top of Form

**Amino acid composition:** 
Ala (A) 1 7.7%

Arg (R) 0 0.0%

Asn (N) 0 0.0%

Asp (D) 5 38.5%

Cys (C) 0 0.0%

Gln (Q) 0 0.0%

Glu (E) 1 7.7%

Gly (G) 2 15.4%

His (H) 0 0.0%

Ile (I) 0 0.0%

Leu (L) 1 7.7%

Lys (K) 0 0.0%

Met (M) 1 7.7%

Phe (F) 2 15.4%

Pro (P) 0 0.0%

Ser (S) 0 0.0%

Thr (T) 0 0.0%

Trp (W) 0 0.0%

Tyr (Y) 0 0.0%

Val (V) 0 0.0%

Pyl (O) 0 0.0%

Sec (U) 0 0.0%

(B) 0 0.0%

(Z) 0 0.0%

(X) 0 0.0%

Bottom of Form

**Total number of negatively charged residues (Asp + Glu):** 6

**Total number of positively charged residues (Arg + Lys):** 0

**Atomic composition:**

Carbon C 61

Hydrogen H 83

Nitrogen N 13

Oxygen O 26

Sulfur S 1

**Formula:** C_61_H_83_N_13_O_26_S_1_

**Total number of atoms:** 184

**Extinction coefficients:** As there are no Trp, Tyr or Cys in the region considered, your protein

should not be visible by UV spectrophotometry.

**Estimated half-life:**The N-terminal of the sequence considered is E (Glu).The estimated half-life is:

- 1 hours (mammalian reticulocytes, in vitro).
- 30 min (yeast, in vivo).
- >10 hours (Escherichia coli, in vivo).

**Instability index:** The instability index (II) is computed to be 11.72. This classifies the protein as stable.

**Aliphatic index:** 37.69

**Grand average of hydropathicity (GRAVY):** -0.669

| [**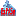ExPASy Home page**](http://www.expasy.ch/) | [**Site Map**](http://www.expasy.ch/sitemap.html) | [**Search ExPASy**](http://www.expasy.ch/ExpasyHunt/) | [**Contact us**](http://www.expasy.ch/contact.html) | [**Proteomics tools**](http://www.expasy.ch/tools/) | [**Swiss-Prot**](http://www.expasy.ch/sprot/) |
| --- | --- | --- | --- | --- | --- |


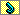

Supplement: File S4 — Showing the Biophysical profiles of the acidic C-terminus of the T.cruzi ribosomal P protein TcP2β. The file provides details of the biophysical profiles, including chemical structure, amino acid composition, Theoretical PI, Instability index, Extinction coefficient Aliphatic Index and Graavy of the C-terminus peptides of TcP2β (EEEDDDMGFGLFD). The data was generated by the Expasy software Protparam [25] (0.24 MB DOC) [file pone.0007420.s005.doc]
